# Smad3‐mediated lncRNA *HSALR1* enhances the non‐classic signalling pathway of TGF‐β1 in human bronchial fibroblasts by binding to HSP90AB1

**DOI:** 10.1002/ctm2.1292

**Published:** 2023-06-14

**Authors:** Erkang Yi, Biting Lin, Yi Zhang, Xiaoyu Wang, Jiahuan Zhang, Yu Liu, Jing Jin, Wei Hong, Zhiwei Lin, Weitao Cao, Xinyue Mei, Ge Bai, Bing Bing Li, Yumin Zhou, Pixin Ran

**Affiliations:** ^1^ Guangzhou Institute of Respiratory Health & State Key Laboratory of Respiratory Disease & National Clinical Research Center for Respiratory Disease & National Center for Respiratory Medicine The First Affiliated Hospital of Guangzhou Medical University Guangzhou Guangdong China; ^2^ GMU‐GIBH Joint School of Life Sciences Guangzhou Medical University Guangzhou Guangdong China; ^3^ Guangzhou Laboratory Bioland Guangzhou Guangdong China

**Keywords:** Akt pathway, cell proliferation, chronic obstructive pulmonary disease, heat shock protein AB1, lncRNA HSALR1, Smad3

## Abstract

**Background:**

Chronic obstructive pulmonary disease (COPD) is one of the diseases with high mortality and morbidity with complex pathogenesis. Airway remodeling is an unavoidable pathological characteristic. However, the molecular mechanisms of airway remodeling are incompletely defined.

**Methods:**

lncRNAs highly correlated with transforming growth factor beta 1(TGF–β1) expression were chosen, the lncRNA *ENST00000440406* (named HSP90AB1 Assoicated LncRNA 1, *HSALR1*) was chosen for further functional experiments. Dual luciferase and ChIP assay were used to detect the upstream of HSALR1, transcriptome sequencing, Cck–8, Edu, cell proliferation, cell cycle assay, and WB detection of pathway levels confirmed the effect of HSALR1 on fibroblast proliferation and phosphorylation levels of related pathways. Mice was infected with adeno–associated virus (AAV) to express *HSALR1* by intratracheal instillation under anesthesia and was exposure to cigarette smoke, then mouse lung function was performed and the pathological sections of lung tissues were analyzed.

**Results:**

Herein, lncRNA *HSALR1* was identified as highly correlated with the TGF–β1 and mainly expressed in human lung fibroblasts. HSALR1 was induced by Smad3 and promoted fibroblasts proliferation. Mechanistically, it could directly bind to HSP90AB1 protein, and acted as a scaffold to stabilize the binding between Akt and HSP90AB1 to promote Akt phosphorylation. In vivo, mice expressed *HSALR1* by AAV was exposure to cigarette smoke (CS) for COPD modeling. We found that lung function was worse and airway remodeling was more pronounced in HSLAR1 mice compare to wild type (WT) mice.

**Conclusion:**

Our results suggest that lncRNA *HSALR1* binds to HSP90AB1 and Akt complex component, and enhances activity of the TGF–β1 smad3–independent pathway. This finding described here suggest that lncRNA can participate in COPD development, and *HSLAR1* is a promising molecular target of COPD therapy.

## Graphical abstract

The study provides the evidence that a novel lncRNA *HSALR1* is highly expressed in chronic obstructive pulmonary disease and is associated with human bronchial fibroblasts proliferation. *HSALR1* acts as a scaffold and promotes stabilization of the HSP90AB1‐CDC37‐Akt protein complex. *HSALR1*‐expressing mice by intratracheal injection of adeno‐associated virus showed increasing severity of COPD after cigarette smoke exposure.

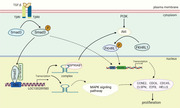

## 1 INTRODUCTION

COPD, one of the diseases with high mortality and morbidity, is a clinically preventable and treatable disease characterized by persistent incomplete reversible disruption of airflow.^1^, ^2^ Its occurrence and development are mainly due to continuous exposure to various risk factors, such as cigarette smoking, biomass smoke, inhalation of traffic exhaust and other particle materials.^3^, ^4^ The pathogenesis of COPD is very complex, with several mechanisms such as airway inflammation, oxidative stress response and protease/antiprotease imbalance implicated in its occurrence.^5^ However, a previous study reported that airway remodelling is an unavoidable pathological characteristic, especially the abnormal fibroblast proliferation.^6^ In addition, TGF‐β1^7^ affects tissue remodelling and proliferation of fibroblasts and smooth muscle cells.^8^

Studies have shown that TGF‐β1 expression is upregulated in the lungs and primary cells (fibroblasts) of COPD patients, suggesting that TGF‐β1 affects COPD development and progression.^9^, ^10^ Cigarette smoke and concomitant smoke‐induced inflammation can induce the production and release of TGF‐β1,^11^ suggesting that TGF‐β1 is a potential therapeutic target for COPD treatment. However, the mechanism underlying TGF‐β1 functions in the body is complex, and only its Smads‐dependent canonical and non‐canonical pathways (MAPK, PI3K/Akt) are extensively studied.^12^ Study has shown that heat shock protein 90 alpha family class B member 1 (HSP90AB1) is a molecular chaperone that can directly stabilize p‐Akt by interacting with p‐Akt.^13^

The emergence of new research element agents, such as long non‐coding RNA (lncRNA), has enhanced the study of the fuelucinctional mechanism of TGF‐β1 and its downstream pathway. LncRNAs have a length longer than 200 nucleotides and lack protein‐coding capacity.^14^ Multiple studies have shown that they perform diverse functions in shaping cellular physiology, structure and function by regulating transcription of critical genes, moulding epigenetics, influencing signalling pathways and modulating protein interactions,^15^ thereby inducing disease‐promoting effects during COPD development.^16^ In our previous transcriptomics analysis,^17^ it was found that lncRNA expression profiles are significantly different between non‐COPD and COPD patients. Moreover, another study showed that lncRNA *IL6‐AS1* is highly expressed in patients with COPD and is closely associated with IL‐6, which provides evidence that lncRNA can be involved in the occurrence and development of COPD.^18^

However, to date, the mechanisms of TGF‐β and its non‐classical pathways have not yet been fully elucidated. Besides, it is unclear whether lncRNA plays an important role in TGF‐β functions. This study used RNA‐sequencing and lncRNA‐mRNA co‐expression network of lung tissues and identified an lncRNA *ENST00000440406.2* (named *HSALR1*) which was highly correlated with TGF‐β expression. Mechanistically, the Smad3 transcription factor positively regulated *HSALR1*, and it also directly interacted with HSP90AB1, thereby enhancing the stabilization between HSP90AB1 and p‐AKT, ultimately leading to continuous activation of the AKT signalling pathway, and the above effect mediates the proliferation of fibroblasts downstream of the AKT pathway. Furthermore, our in vivo animal experiments have provided further confirmation that *HSALR1* is involved in the onset and progression of COPD. This was demonstrated through intratracheal instillation of adeno–associated virus coding HSALR1 (AAV‐*HSALR1*). Overall, this study elucidates a mechanism of the lncRNA‐mediated non‐canonical pathway of TGF‐β signalling, which partially reveals the mechanism of COPD, and thus it might be one of the potential therapeutic targets in the future.

## 2 RESULTS

### 2.1 lncRNA *HSALR1*, regulated by TGF‐β, was expressed in lung fibroblasts

**FIGURE 1:**
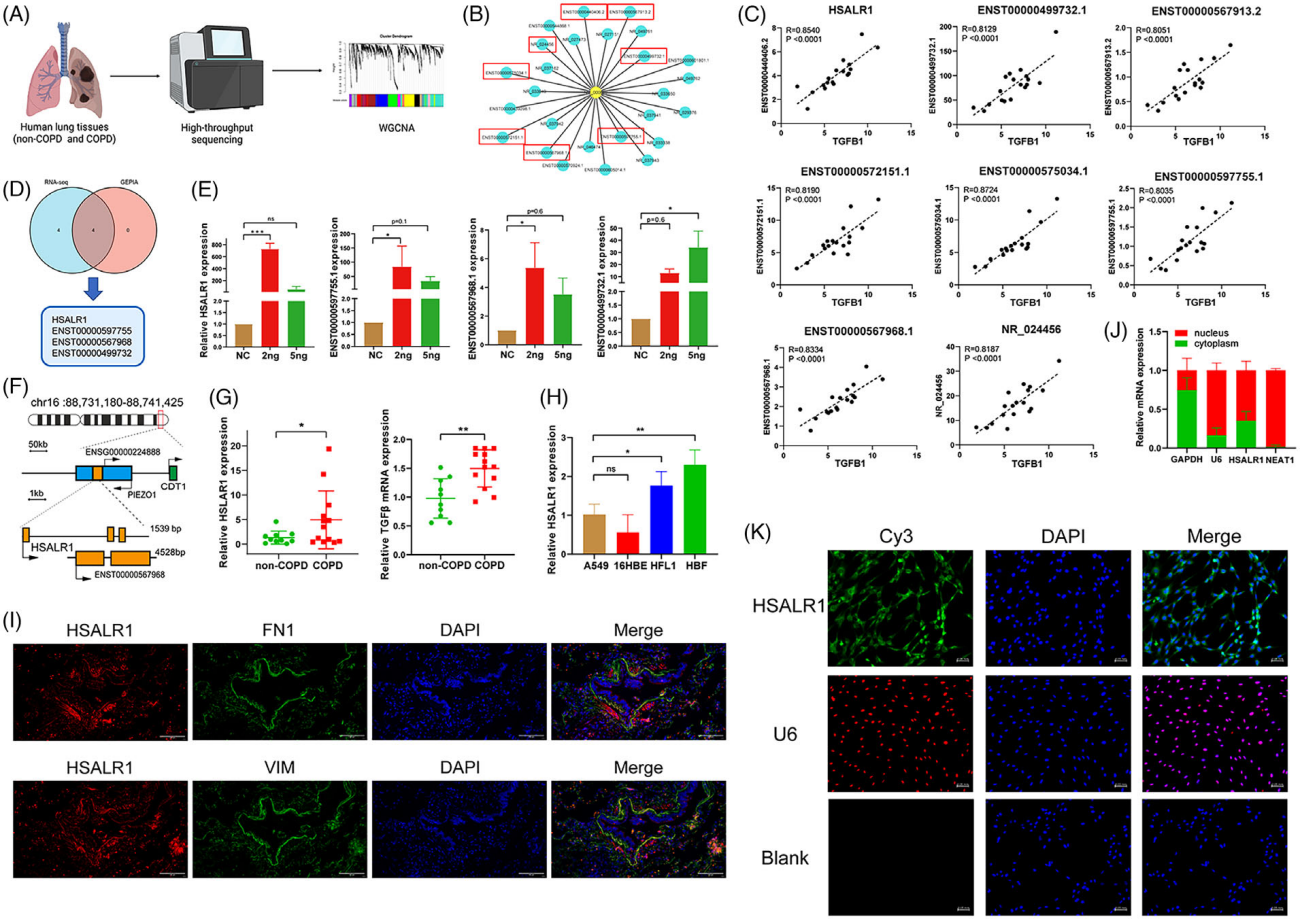
lncRNA *HSALR1* was highly correlated with TGF‐β in lung tissues and was expressed mostly within the fibroblast. (A) Schematic representation of RNA‐seq and co‐expression modules via WCGNA. (B) Schematic representation of the top 25 lncRNAs highly correlated with TGF‐β expression in RNA‐seq of human lung tissues. (C) Correlation analysis between the expression of top eight most correlated lncRNAs and TGF‐β expression in RNA‐seq results. (D) The intersection of the Venn diagram showing the overlapping genes (*HSALR1*, *ENST00000587755*, *ENST00000567968* and *ENST00000499732*) between both correlated lncRNAs in RNA‐seq and GEPIA2. (E) qRT‐PCR analysis of the expression of *HSALR1*, *ENST00000597755*, *ENST00000567968* and *ENST00000499732* (*HSALR1*, *lnc‐10*, *lnc‐12* and *lnc‐15* in graphs) after TGF‐β stimulation (0, 2.5, 5 ng/mL) in HBF cells for 48 h (*n* = 3 biological replicates, one‐way ANOVA). (F) Schematic indicating the genomic location of lncRNA *HSALR1*. (G) qRT‐PCR analysis of the *HSALR1* and TGF‐β in samples from 10 non‐chronic obstructive pulmonary disease (non‐COPD) and 13 COPD smokers (Student's *t*‐test). (H) qRT‐PCR analysis of *HSALR1* expression in various airway structure cells, including A549, 16HBE, HFL1 and HBF (one‐way ANOVA, *n* = 4 biological replicates). (I) Colocalization analysis: RNA FISH assay of *HSALR1* combined with immunofluorescence detection of FN1/Vimentin in human lung tissue sections. Red, HSLAR1; Green, FN1/Vimentin; Blue, DAPI (*n* = 3 replicates, the number of images observed = 3). (J) Nuclear fractionation analysis and qRT‐PCR analysis of *HSALR1* expression in the nucleus and cytoplasm (*n* = 3 biological replicates). (K) FISH assay showing the subcellular localization of *HSALR1* in HBFs. U6 was used as a positive control for nuclear localization; Blank was used as the blank control with negative probes. Green, HSLAR1; Red, U6; Blue, DAPI (*n* = 3 biological replicates, the number of images observed = 3). Data information: Error bars represent means ± SD. *p < .05, **p < .01 and ***p < .05.

In this study, we established lncRNA‐mRNA co‐expression network via weighted gene co‐expression network analysis (WGCNA, Figure 1A) and various lncRNAs that are highly correlated with TGF‐β1 expression (correlation coefficient > 0.8, Figure 1B and Table S1) based on the previous sequencing results of lung tissues samples from non‐COPD and COPD patients.^17^ Results showed that 8 of the 25 lncRNAs, including *HSALR1*, *ENST00000597755.1*, *ENST00000601801.1*, *ENST00000567968.1*, *ENST00000572151.1*, *NR_024456*, *ENST00000499732.1* and *ENST00000567913.2*, were specifically amplified in lung tissues. Moreover, there was a correlation between TGF‐β1 expression and the eight lncRNAs selected through GEPIA database in normal lung tissues^19^ (Figure S1). Results showed that *HSALR1*, *ENST00000597755.1*, *ENST00000567968.1* and *ENST00000499732.1* were significantly associated with TGF‐β1 expression (Figure 1C) and thus they were selected for further experiments (Figure 1D).

**FIGURE 2:**
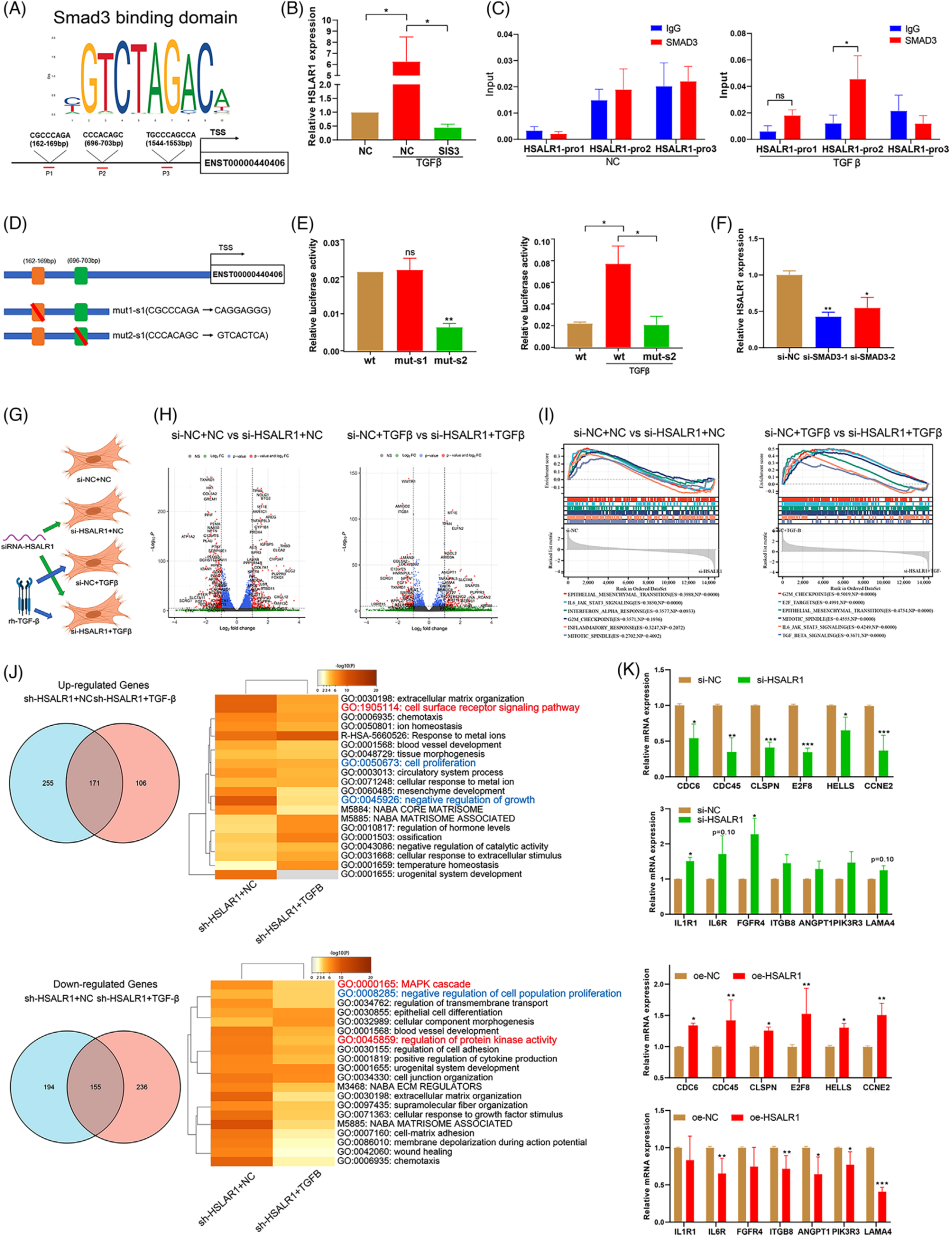
HSALR1 was regulated by Smad3 and was seem to affect PI3K/Akt pathway and cell proliferation through RNA‐seq. (A) Schematic of the binding motif of transcription factor Smad3, and three transcription factor binding sites (162–169, 696–703 and 1544–1553 bp) of Smad3 predicted on lnc9 promoter using JASPAR (http://jaspar.genereg.net/). The red arrow indicates the EBF1 transcription factor. (B) qRT‐PCR analysis of lnc9 expression after treatment with TGF‐β or SIS3 + TGF‐β (*n* = 3 biological replicates, Student's *t*‐test). (C) ChIP‐PCR analysis of Smad3 occupancy on *HSALR1* promoter in unstimulated HBF cells and TGF‐β‐stimulated HBF cells. IgG was used as the negative control (*n* = 3 biological replicates, Student's *t*‐test). (D) Schematic representation of the two mutation sequences of potential Smad3‐binding sites on *HSALR1* promoter. (E) Luciferase activity in the *HSALR1* promoter after transfection with a reporter containing wild‐type or mutant *HSALR1* promoter in unstimulated HBF cells and TGF‐β‐stimulated HBF cells with TGF‐β (*n* = 3 biological replicates, Student's *t*‐test). (F) qRT‐PCR analysis of *HSALR1* expression after transfection with Smad3 siRNA in HBF cells (*n* = 3 biological replicates, one‐way ANOVA). (G) Schematic representation of the cell samples divided into four groups (si‐NC + NC, si‐*HSALR1* + NC, si‐NC + TGF‐β and si‐*HSALR1* + TGF‐β) for RNA‐seq (*n* = 3 biological replicates). (H) The volcano plot of the DEGs between si‐NC + NC and si‐*HSALR1* + NC/si‐NC + TGF‐β and si‐*HSALR1* + TGF‐β (*n* = 3 biological replicates). (I) Results of GSEA analysis of RNA sequencing by using Hallmark pathway database. (J) The intersection of the Venn diagram showing the upregulated and downregulated overlapping genes between both comparison DEGs in si‐NC + NC and si‐lnc9 + NC/si‐NC + TGF‐β and si‐lnc9 + TGF‐β; and enrichment analysis of DEGs in overlapping genes. (K) qRT‐PCR analysis of proliferation‐associated genes (*CDC6, CDC45, CCNE2, E2F8, CLSPN*, etc.) and cytokine‐associated genes (*IL1R1, IL6R, FGFR4, IGTB8, ANGPT1, PIK3R3, LAMA4*, etc.) after *HSALR1* knockdown using siRNA or *HSALR1* overexpression using overexpression lentiviral vector in HBF cells (*n* = 4 biological replicates, Student's *t*‐test). Data information: Error bars represent means ± SD. *p < .05, **p < .01 and ***p < .05.

Different structural cells in lungs, including HBFs, A549 and 16 HBE cells, were selected for in vitro experimental modelling to determine whether the four lncRNAs are upstream or downstream of TGF‐β1. In these three types of cells, we observed that cell viability remained unchanged when exposed to 2.5 and 5 ng/mL TGF‐β1 (Figure S2A). Subsequently, cells were first stimulated with these two concentrations and then the expression of the four lncRNAs was measured. Results indicated that the expression of lncRNAs significantly changed in fibroblasts (Figure 1E) compared to 16HBE (Figure S2B) and A549 (Figure S2C) cells. Knockdown of the four lncRNAs using specific siRNA did not significantly affect TGF‐β1 expression (Figure S2D). While the expression of *HSALR1* was notably elevated in HBFs following exposure to TGF‐β1, the expression of TGF‐β1 was not affected, suggesting that *HSALR1* is a target downstream of TGF‐β1. Moreover, the AnnoLnc2 database^20^ revealed that the expression of *HSALR1* was the most significantly increased in colon tissues, followed by lung tissues (Figure S3A). The PhyloCSF analysis indicated that *HSALR1* had no protein‐coding potential (Figure S3B). The Ensembl database showed that *HSALR1* maps to chromosome 16 q24.3 and has three exons that are 1539 bp long (Figure 1F). Furthermore, our findings demonstrated that both *HSALR1* and TGF‐β1 exhibited higher RNA expression levels in individuals with COPD compared to those without the condition *HSALR1* (Figure 2G). Therefore, *HSALR1* was chosen as the candidate gene for subsequent functional studies.

Studies have reported that the way an lncRNA exerts its function mainly depends on its specifically expressed cell type and subcellular localization.^21^, ^22^ Herein, qRT‐PCR analysis results in different types of cells showed that *HSALR1* was mainly expressed in fibroblast (Figure 1H), the finding above was also supported by a combination of fluorescence in situ hybridization (FISH) and immunofluorescence staining assay with (Figure 1I). In Human bronchial fibroblasts (HBFs), *HSALR1* was present in both the nuclei and cytoplasm (Figure 1J). Furthermore, the outcomes of the nuclear–cytoplasmic separation assays conducted on HBFs were in agreement with the results of the FISH assay (Figure 1K). Combining the results above, *HSALR1* was selected for further functional study.

### 2.2 HSALR1 regulated by TGF‐β/smad3 may affect Akt/MAPK pathway‐related genes

Numerous studies have reported that TGF‐β1 acts through the classic TGF‐β1/Smads pathway.^23^ In this study, we found three potential Smad3‐binding sites (P1, P2 and P3) in the *HSALR1* promoter region using the JASPAR database (Figure 2A). RNA‐seq results and the GEPIA database (Figure S4A,B) showed that Smad3 was positively correlated with *HSALR1* in lung tissues. We further analysed online database to verify the accuracy of the aforementioned data. Results showed that the SIS3 inhibitor significantly alleviated the upregulation of *HSALR1* caused by TGF‐β1 (Figure 2B).

The chromatin immunoprecipitation (ChIP) assay was performed using anti‐Smad3 antibody, with obtained results showing that Smad3 could directly bind to the *HSALR1* promoter under TGF‐β1 stimulation (Figure 2C). Furthermore, to determine the exact Smad3‐binding site in the *HSALR1* promoter, two luciferase reporters were constructed through deletion of putative Smad3‐binding sites based on the ChIP–qPCR results (*HSALR1*‐pro1/mut1 and *HSALR1*‐pro2/mut2, Figure 2D). The study findings indicated that the mutation of the Smad3‐binding site 2 (162–169 bp) resulted in a reduction in the basal luciferase activity of the *HSALR1* promoter. However, the mutation of Smad3‐binding site 1 (696–703 bp) did not have any impact on the promoter activity. Moreover, the mutant of Smad3‐binding site 2 significantly attenuated the increased *HSALR1* promoter activity due to TGF‐β1 stimulation (Figure 2E). In addition, knockdown of Smad3 with two specific siRNAs (Figure S4C,D) significantly decreased *HSALR1* expression (Figure 2F). Moreover, FISH assay showed that the fluorescence intensity of *HSALR1* in the nucleus was increased after TGF‐β1 stimulation, which indicated TGF‐β1 promoted the nuclear transcription of *HSALR1* in HBFs (Figure S5). Collectively, these results suggest that TGF‐β1 can upregulate *HSALR1* expression through the classical TGF‐β1/Smad3 pathway.

To explore potential functions of *HSALR1* in HBFs, samples were first divided into four groups (si‐NC + NC, si‐*HSALR1* + NC, si‐NC + TGF‐β and si‐*HSALR1* + TGF‐β) and then subjected to RNA‐seq (Figure 2G). RNA‐seq results identified 349 significantly upregulated and 426 significantly downregulated genes in si‐NC + NC versus si‐*HSALR1* + NC, and 391 significantly upregulated and 277 significantly downregulated genes in si‐NC + TGF‐β versus si‐*HSALR1* + TGF‐β (Figure 2H). Gene set enrichment analysis (GSEA) results suggested that *HSALR1* might participate in the proliferation‐associated and inflammation‐associated pathway (Figure 2I). Furthermore, a total of 155 genes were upregulated and 171 genes were downregulated in common (i.e., overlapping genes) between si‐NC + NC/si‐*HSALR1* + NC and si‐NC + TGF‐β/si‐*HSALR1* + TGF‐β. Subsequently, KEGG and GO enrichment analyses showed that the overlapping genes were mainly involved in cell proliferation, negative regulation of growth, MAPK cascade and protein kinase activity (Figure 2J). Moreover, it was found that TGF‐β1 stimulation upregulated 47 genes, but *HSALR1* knockdown alleviated the effect. TGF‐β1 stimulation also downregulated 103 genes, and the effect was alleviated via overexpression of *HSALR1* (Figure S6A–C and Table S3). Similarly, KEGG and GO enrichment analyses showed that the genes were enriched in the PI3K‐AKT pathway and cell cycle, indicating that *HSALR1* can influence activation of the PI3K‐AKT pathway and cell proliferation (Figure S6D,E). Subsequently, we generated an lnc9‐overexpression vector to induce the overexpression of *HSALR1* (oe‐*HSALR1*1; Figure S7A) and two shRNA vectors to knockdown *HSALR1* expression (sh‐*HSALR1*‐1 and sh‐*HSALR1*‐2; Figure S7B), followed by qRT‐PCR analysis to detect the expression of proliferation‐associated genes (*CDC6, CDC45, CCNE2, E2F8* and *CLSPN*), PI3K/Akt pathway‐associated genes and cytokine‐associated genes (*IL1R1, IL6R, FGFR4, IGTB8, ANGPT1, PIK3R3* and *LAMA4*). The qRT‐PCR results demonstrated that *HSALR1* was capable of regulating a majority of the proliferation‐associated genes and cytokine‐associated genes *HSALR1* (Figure 2K). Taken together, these results suggest that *HSALR1* is involved in proliferation of HBFs and the PI3K/Akt pathway.

### 2.3 *HSALR1* acts as a scaffold and stabilizes the HSP90AB1‐Akt1 complex

**FIGURE 3:**
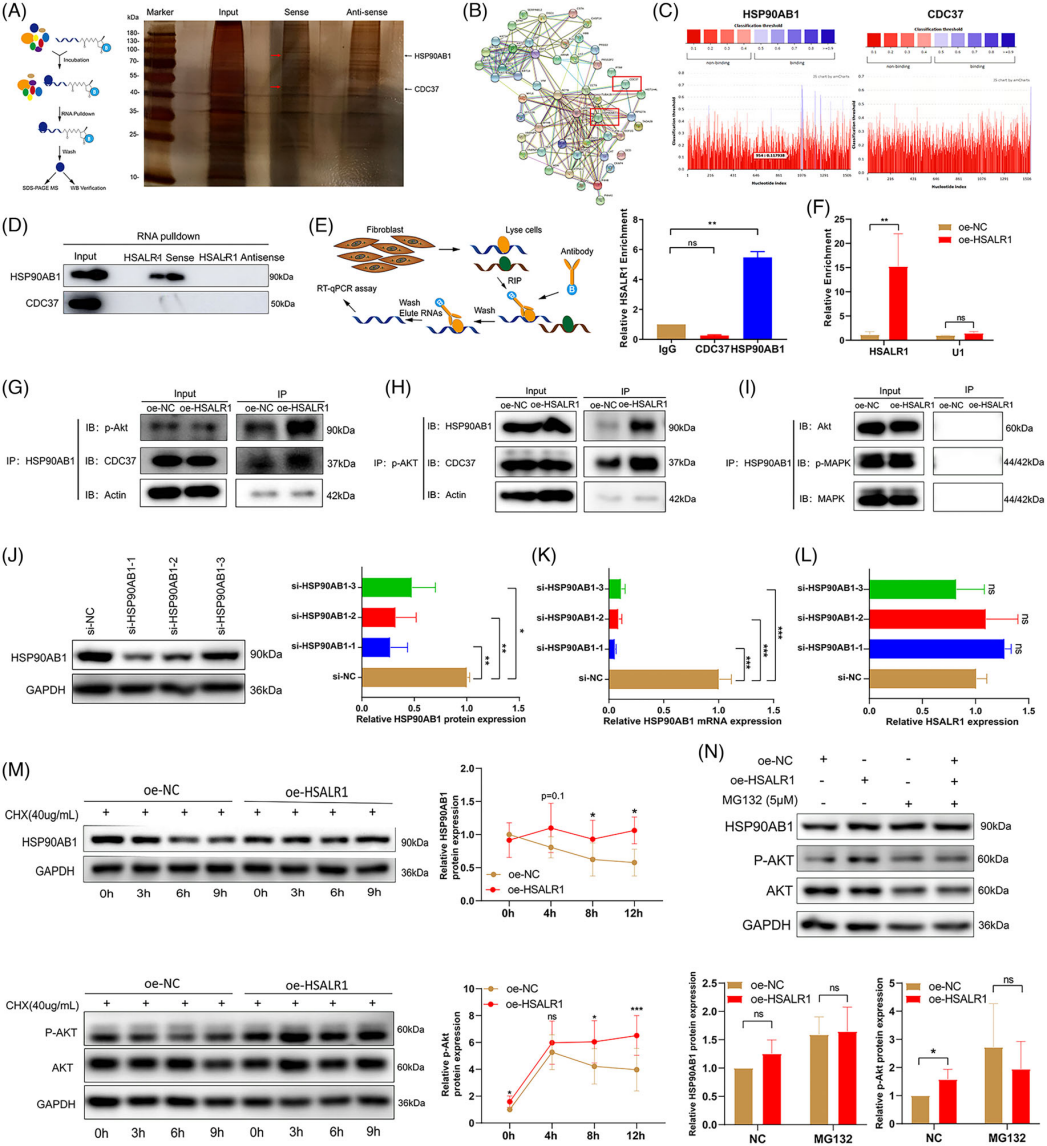
HSALR1 acts as a scaffold and stabilizes HSP90AB1‐Akt1 complex. (A) RNA pulldown assay using lncRNA *HSALR1* sense and antisense RNAs in HBF cells, followed by silver staining (*n* = 3 biological replicates). (B) Schematic representation of the PPI network of overlapping genes occurring twice or more in MS. (C) The bindings of *HSALR1* to HSP90AB1 and CDC37 predicted via RNAInter. (D) The interaction between *HSALR1* and HSP90AB1 versus *HSALR1* and CDC37 detected by Western blotting with the extract of the RNA pulldown assay. (E) RIP‐qPCR analysis with anti‐HSP90AB1 antibody and anti‐CDC37 antibody in HBF cells (*n* = 3 biological replicates, Student's *t*‐test). (F) RIP‐qPCR analysis with anti‐HSP90AB1 antibody and anti‐CDC37 antibody after *HSALR1* overexpression in HBF cells (*n* = 3 biological replicates, Student's *t*‐test). (G) Co‐IP analysis of lnc9‐overexpressed HBFs using anti‐HSP90AB1 antibody. Western blot was used to verify the Co‐IP results with anti‐p‐AKT and anti‐CDC37 antibodies. (H) Co‐IP analysis of lnc9‐overexpressed HBFs using anti‐p‐AKT antibody. Western blot was performed to verify the Co‐IP results with anti‐HSP90AB1 and anti‐CDC37 antibody. (I) Co‐IP analysis of lnc9‐overexpressed HBFs using anti‐HSP90AB1 antibody. Western blot was conducted to verify the Co‐IP results with anti‐AKT, anti‐p‐MAPK and anti‐MAPK antibodies. (J) Western blot and (K) qRT‐PCR analysis of HSP90AB1 expression after transfection with three siRNAs (siHSP90AB1‐1, si‐HSP90AB1‐2 and siHSP0AB1‐3) in HBF cells; and (L) qRT‐PCR analysis of *HSALR1* expression after transfection with above siRNAs (*n* = 3 biological replicates, one‐way ANOVA). (M) 48 h after overexpression of *HSALR1*, the HBFs were incubated with cycloheximide (CHX, 40 μg/mL) and negative control (DMSO), then harvested at 0, 3, 6, 9 h (*n* = 5 biological replicates, one‐way ANOVA). (N) 48 h after overexpression of *HSALR1*, the HBFs were incubated with MG‐132 (5 μm) and negative control (DMSO), then harvested at 12 h (*n* = 5 biological replicates, Student's *t*‐test). Data information: Error bars represent means ± SD. *p < .05, **p < .01 and ***p < .05.

Studies have shown that lncRNAs exert their regulatory function by binding to proteins.^24^ This study conducted three independently repeated RNA pulldown and mass spectrometry assays to further explore the functions of *HSALR1* (Figure 3A). The STRING database was then utilized to construct a protein–protein interaction (PPI) network using genes that appeared more than twice in MS results (Figure 3B). Notably, the PPI network had HSP90AB1 and CDC37, and HSP90AB1 was identified as the hub gene using cytoHubba (Figure S7C,D). Next, HSP90AB1 and CDC37 proteins located in the specific protein band were used for further analysis to detect the potential target proteins of *HSALR1*. Moreover, RNAInter^25^ was applied to predict the interaction among HSP90AB1, CDC37 and *HSALR1*. Results showed that there was a significant interaction between HSP90AB1 and *HSALR1* (Figure 3C). Western blot analysis only detected HSP90AB1 in the *HSALR1* pulldown complex (Figure 3D). Similarly, the RNA immunoprecipitation (RIP) assay revealed that *HSALR1* strongly interacted with HSP90AB1 compared to the negative control (IgG) (Figure 3E). The RIP results also showed that overexpression of *HSALR1* increased *HSALR1* enrichment in HBFs by using the HSP90AB1 antibody (Figure 3F).

Previous studies have shown that HSP90AB1 plays a crucial role in the non‐classical TGF‐β pathway,^26^ including binding to Akt for maintaining its kinase activity. Herein, overexpression or knockdown of *HSALR1* did not exert any additional effects on the RNA or protein expression of HSP90AB1 (Figure S7E,F). The enrichment and RNA pulldown results suggest that *HSALR1* might act as a scaffold to promote and stabilize the interaction between HSP90AB1 and Akt. Moreover, Co‐immunoprecipitation (Co‐IP) assay using anti‐HSP90AB1 antibody showed that overexpression of *HSALR1* enhanced the binding efficiency of HSP90AB1 to p‐Akt and HSP90AB1 to CDC37 (Figure 3G). Similarly, the results demonstrated that more p‐Akt protein was bound to HSP90AB1 and CDC37 in overexpression samples compared to the negative control (Figure 3H). However, HSP90AB1 did not bind to total Akt, total MAPK and p‐MAPK protein (Figure 3I), suggesting that HSP90AB1 only works on the phosphorylated Akt (p‐Akt) protein in HBFs.

To determine whether HSP90AB1 affected the stability of *HSALR1*, HSP90AB1 was knocked down using three specific siRNAs (Figure 3J,K) and the obtained results showed that HSP90AB1 had no effect on stability of *HSALR1* (Figure 3L). Moreover, overexpression of *HSALR1* in HBFs displayed higher protein stability of HSP90AB1 and p‐Akt in response to CHX treatment (Figure 3M). However, MG132, a proteasome inhibitor, rescued the change in p‐Akt protein levels induced by *HSALR1* overexpression (Figure 3N). Collectively, these results imply that *HSALR1* acts as a scaffold and promotes stabilization of the HSP90AB1‐CDC37‐Akt protein complex.

### 2.4 *HSALR1* promotes proliferation of HBF and activation of TGF‐mediated non‐classical pathways

**FIGURE 4:**
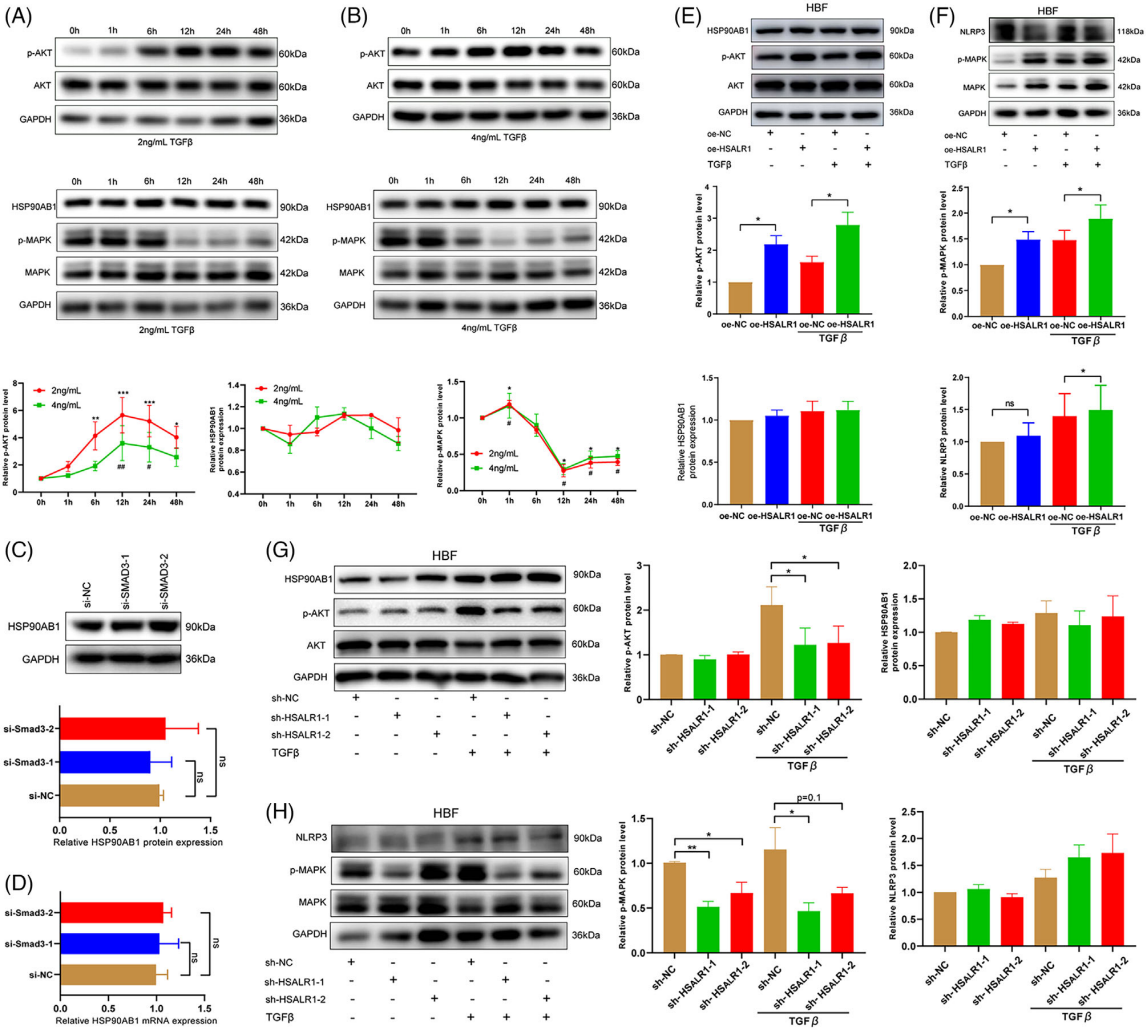
HSALR1 promotes the activation of TGF‐mediated non‐classical pathways. Western blot assays of p‐Akt and p‐MAPK with GAPDH as the control after HBFs stimulation with TGF‐β [(A) 2 ng/mL and (B) 4 ng/mL] at various time points (0, 1, 6, 12 and 24 h) (*n* = 3 biological replicates, one‐way ANOVA). (C,D) Western blot and qRT‐PCR showing the expression of HSP90AB1 after knockdown of Smad3 by siSmad3‐1 and siSmad3‐2 (*n* = 3 biological replicates, Student's *t*‐test). (E) Western blot assay results showing the expression of HSP90AB1 and activation level of the Akt signalling pathway after overexpression of *HSALR1* and stimulation 12 h by TGF‐β (2 ng/mL) (*n* = 4 biological replicates, Student's *t*‐test). (F) Western blot indicating the expression of NLRP3 and the activation level of the MAPK signalling pathway after *HSALR1* overexpression and stimulation 1 h by TGF‐β (2 ng/mL) (*n* = 4 biological replicates, Student's *t*‐test). (G) Western blot showing the expression of HSP90AB1 and the activation level of the Akt signalling pathway after *HSALR1* knockdown and stimulation 12 h by TGF‐β (2 ng/mL) (*n* = 4 biological replicates, Student's *t*‐test). (H) Western blot showing the expression level of NLRP3 and the activation level of the MAPK signalling pathway after *HSALR1* knockdown and stimulation 1 h by TGF‐β (2 ng/mL) (*n* = 4 biological replicates, Student's *t*‐test). Data information: Error bars represent means ± SD. *p < .05, **p < .01 and ***p < .05.

Many studies have reported on the non‐classical TGF‐β pathways, including MAPK and PI3K/Akt pathways.^27^ Results obtained in this study showed that phosphorylation of TGF‐β‐induced Akt reached its optimal amounts after 6 or 12 h of stimulation, whereas MAPK phosphorylation reached its optimal amounts after only 1 h of stimulation, but the extent of phosphorylation was decreased after 12 h of stimulation (Figure 4A,B). However, no significant changes were detected in HSP90AB1 protein level after TGF‐β stimulation (Figure 4A,B), and Smad3 knockdown had no effect on the protein and RNA level of HSP90AB1 (Figure 4C,D). The findings indicate that the upregulation of HSP90AB1 expression does not mediate the activity of non‐classical TGF‐β pathways, and the expression of HSP90AB1 does not activate the TGF‐β/Smad3 pathway. Therefore, we speculated that HSALR rather than HSP90AB1 is responsible for the activity of the Akt and MAPK pathway. Next, we assessed levels of p‐Akt and phosphorylated MAPK (p‐MAPK) after overexpression or knockdown of *HSALR1* in HBFs. Although *HSALR1* did not affect HSP90AB1 expression under no‐stimulation/TGF‐β‐stimulation condition, overexpression of *HSALR1* increased the levels of p‐Akt and MAPK (Figure 4E,F). In contrast, *HSALR1* knockdown decreased Akt and MAPK activity, but did not affect HSP90AB1 expression (Figure 4G,H). Furthermore, the above experiment was repeated on another fibroblast (HFL1) using the same conditions, and similar results were found (Figure S8A,B). Prior studies have indicated that HSP90AB1 can bind to NLRP3 and Act1.^28^, ^29^ However, our findings revealed that overexpression of *HSALR1* did not impact the expression of NLRP3 nor activate the NF‐κB pathway *HSALR1* (Figure S8C,D Supporting Information). Moreover, overexpression or knockdown of *HSALR1* did not significantly affect the RNA levels of the above pathway‐related genes (Figure S9A‐H), indicating that *HSALR1* regulates the Akt and MAPK pathway without affecting the RNA level of the genes. Results from the GEPIA database indicated that *HSALR1* expression was significantly correlated with Akt1 and *MAPK1* expression, but was not correlated with the expression of *HSP90AB1*, *NLRP3*, *NFKB1* and *RELA* in lung tissues (Figure S9I,J). These results suggest that TGF‐β/Smad3‐regulated *HSALR1* can regulate the non‐classical TGF‐β pathway and MAPK pathway.

**FIGURE 5:**
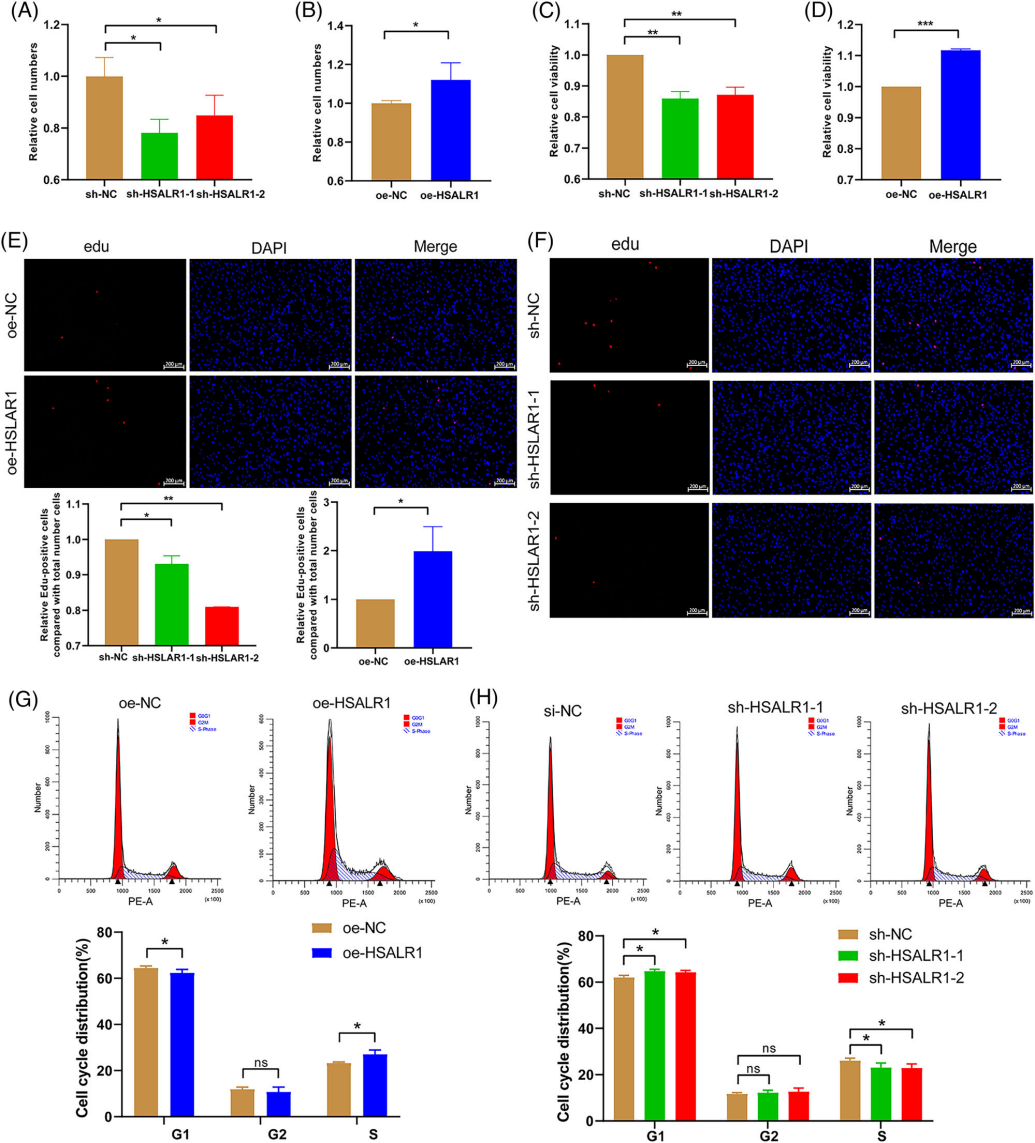
HSALR1 promotes HBFs proliferation. Cell count of the control and (A) *HSALR1* knockdown or (B) *HSALR1* overexpression HBF cells (*n* = 3 biological replicates, Student's *t*‐test). Cell counting kit‐8 (CCK‐8) assay of the control and (C) *HSALR1* knockdown or (D) *HSALR1* overexpression HBF cells (*n* = 3 biological replicates, Student's *t*‐test). Edu assay of the control and (E) *HSALR1* knockdown or (F) *HSALR1* overexpression HBF cells (*n* = 4 biological replicates, Student's *t*‐test). Cell cycle flow cytometry and PI staining assessing the control and (G) *HSALR1* knockdown or (H) *HSALR1* overexpression HBF cells (*n* = 4 biological replicates, Student's *t*‐test). Data information: Error bars represent means ± SD. *p < .05, **p < .01 and ***p < .05.

RNA‐seq results revealed that *HSALR1* knockdown downregulated the proliferation‐associated genes, which indicate that cell cycle progression is downstream of the PI3K/Akt and MAPK pathway. This study further constructed two different lentiviruses expressing shRNA for *HSALR1* knockdown with higher knockdown efficiency to investigate whether *HSALR1* participates in the proliferation of fibroblasts. The cell counting assay results showed that *HSALR1* knockdown reduced cell proliferation, whereas *HSALR1* overexpression promoted cell proliferation (Figure 5A,B). Moreover, the cell counting kit‐8 (CCK‐8) assay (Figure 5C,D) and Edu assay (Figure 5E,F) results were consistent with those obtained from the cell counting results. Furthermore, we performed flow cytometric analyses to evaluate the impact of *HSALR1* on cell cycle progression of HBFs. The findings demonstrated that the upregulation of HSALR facilitated the entry of human bronchial fibroblasts (HBFs) into the S‐phase, whereas its downregulation impeded cell entry into the S‐phase (Figure 5G,H). These results suggest that *HSALR1* positively regulates the proliferation of HBFs in vitro.

### 2.5 Smad3‐mediated *HSALR1* promotes HBFs proliferation via the Akt pathway by binding to HSP90AB1

Furthermore, we explored whether Smad3 and HSP90AB1 are involved in activating Akt and MAPK pathways in HBFs. Results showed that siRNA‐mediated knockdown of Smad3 decreased the p‐Akt in HBFs but did not affect the MAPK pathway (Figure S10A), which is consistent with previous observations.^12^ HBFs were further transfected with three distinct siRNA of HSP90AB1 with high interference efficiency. Similarly, results showed that siRNA‐mediated knockdown of HSP90AB1 decreased the p‐Akt and MAPK pathway in HBF (Figure S10B), which is consistent with the findings from a previous study.^30^ We also explored the effects of Smad3 and HSP90AB1 on the above target genes of *HSALR1*, with obtained results showing that knockdown of Smad3 and HSP90AB1 leads to a decrease in the proliferation‐related genes and an increase in the cytokine‐related genes, which were broadly consistent with the functional effect of *HSALR1* (Figure S10C,D).

**FIGURE 6:**
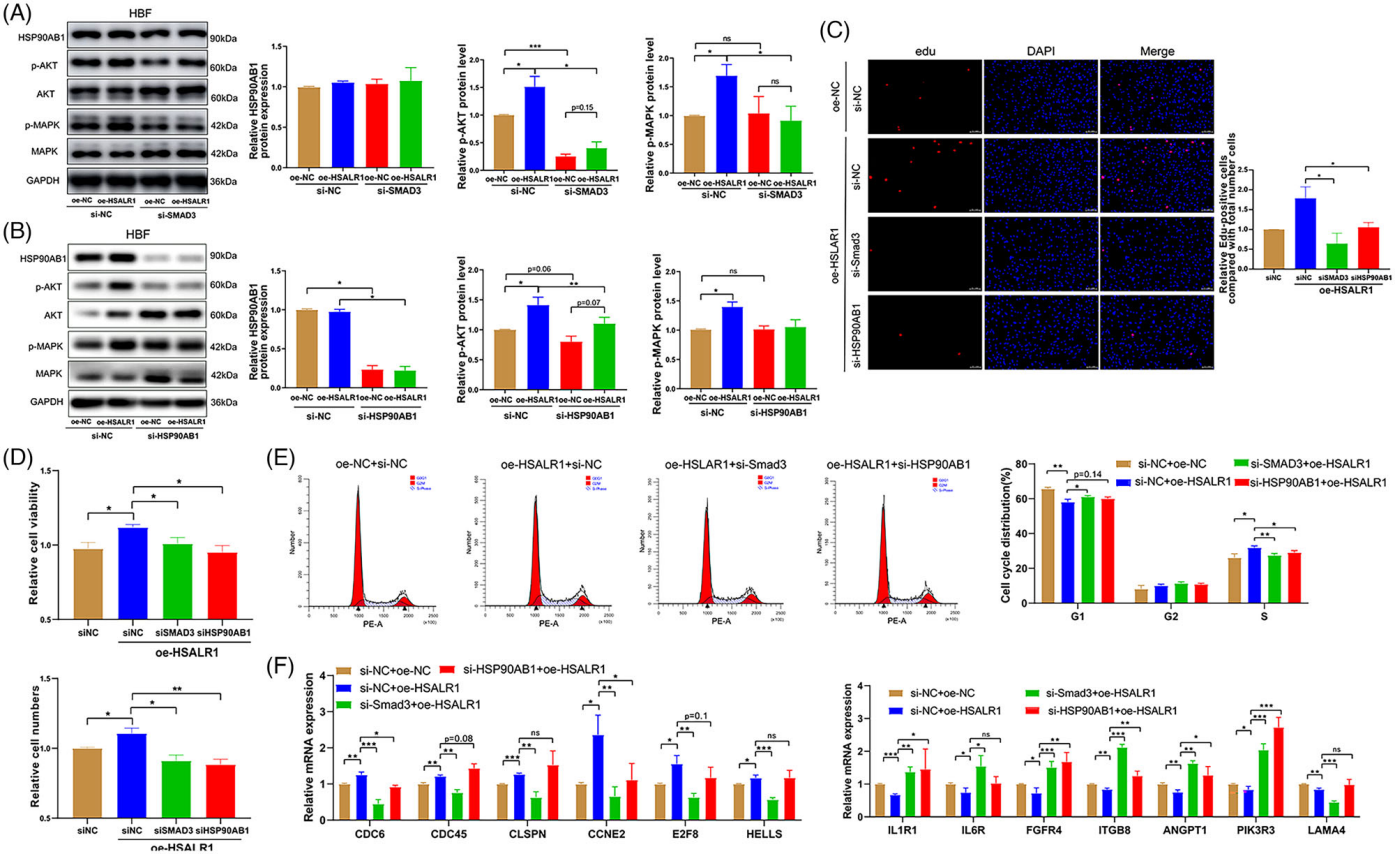
Smad3‐mediated *HSALR1* promotes HBFs proliferation via Akt pathway by binding to HSP90AB1. (A) Western blot showing the expression of HSP90AB1, the activation level of the Akt and MAPK signalling pathway in HBFs after *HSALR1* overexpression and Samd3 knockdown, with GAPDH as the control (*n* = 5 biological replicates, one‐way ANOVA). (B) Western blot showing the expression of HSP90AB1, the activation level of the Akt and MAPK signalling pathway in HBFs after *HSALR1* overexpression, and Smdd3 knockdown with GAPDH as the control (*n* = 5 biological replicates, one‐way ANOVA). (C) Edu assay, (D) cell count assay and CCK‐8 of HBF cells after *HSALR1* overexpression and Samd3 or HSP90AB knockdown (*n* = 4 biological replicates, one‐way ANOVA). qRT‐PCR analysis of above proliferation‐associated genes and cytokine‐associated genes after Smad3 knockdown using two siRNA (si‐Smad3‐1 and si‐Smad3‐2) in HBF cells (*n* = 4 biological replicates, Student's *t*‐test). (D) qRT‐PCR analysis of above proliferation‐associated genes and cytokine‐associated genes after HSP90AB1 knockdown using siRNA (si‐HSP90AB1‐1 and si‐HSP90AB1‐2) in HBF cells (*n* = 4 biological replicates, Student's *t*‐test). (E) Cell cycle Flow cytometry and PI staining assessing the HBF cells after *HSALR1* overexpression and Samd3 or HSP90AB knockdown (*n* = 4 biological replicates, one‐way ANOVA). (F) qRT‐PCR analysis of proliferation‐associated genes (*CDC6*, *CDC45*, *CCNE2*, *E2F8*, *CLSPN*, etc.) and cytokine‐associated genes (*IL1R1*, *IL6R*, *FGFR4*, *IGTB8*, *ANGPT1*, *PIK3R3*, *LAMA4*, etc.) after *HSALR1* overexpression and Samd3 or HSP90AB1 knockdown in HBFs (*n* = 4 biological replicates, one‐way ANOVA). Data information: Error bars represent means ± SD. *p < .05, **p < .01 and ***p < .05.

In addition, this study conducted rescue experiments to provide further validation of the regulatory mechanism involving the Smad3/*HSALR1*/HSP90AB1 axis. Results indicated that overexpression of *HSALR1* promoted the p‐MAPK and PI3K/Akt pathways. However, the co‐transfection of knocked down Smad3 and overexpressed *HSALR1* rescued the activity of the above pathway (Figure 6A). Similar results were obtained after co‐transfection of knocked down HSP90AB1 and overexpressed *HSALR1* (Figure 6B).

In addition, rescue experiments of cell proliferation assays were conducted. The Edu (Figure 6C), cell count and CCK‐8 (Figure 6D) assays indicated that knockdown of Smad3 and HSP90AB1 partially alleviated the *HSALR1*‐induced proliferation of HBFs. Moreover, co‐transfection of the siRNAs of Smad3 and HSP90AB1 partially decreased the S stage of HBFs (Figure 6E). Overexpression of *HSALR1* also upregulated proliferation‐associated genes and downregulated cytokine‐associated genes. However, the above effects were significantly suppressed by Smad3 or HSP90AB1 knockdown (Figure 6F). In summary, the above results demonstrated that the Smad3/*HSALR1*/Akt axis facilitates cell proliferation.

### 2.6 AAV9‐*HSALR1* mice showed more COPD‐like pathological changes after smoke exposure

**FIGURE 7:**
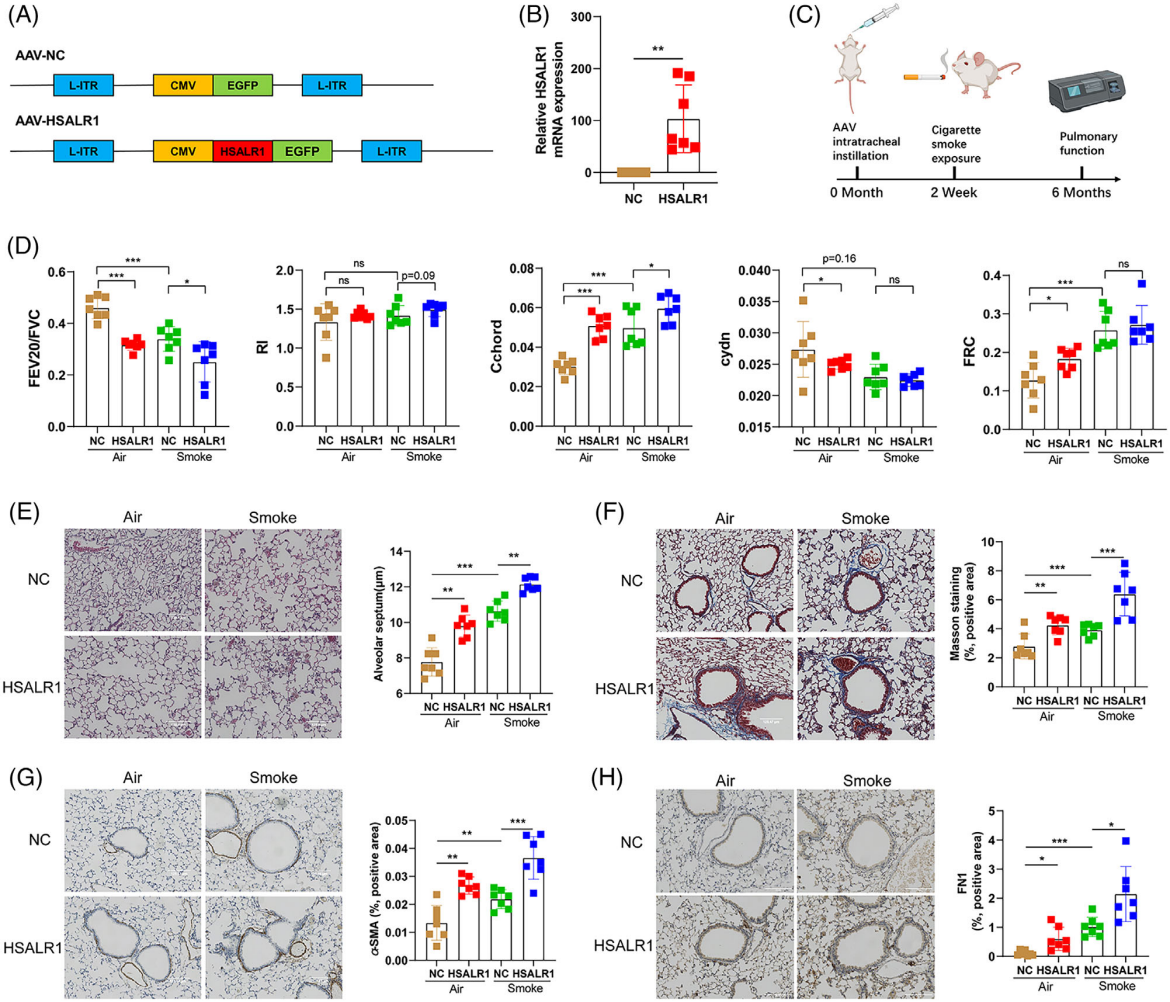
HSALR1 mice showed noticeable pathological differences of chronic obstructive pulmonary disease than wild‐type mice. (A) Schematic structures of AAV‐based negative control vector (AAV‐NC) and AAV‐based *HSALR1* gene vector (AAV‐HSALR). (B) qRT‐PCR analysis of the expression of *HSALR1* between AAV‐NC mice and AAV‐*HSALR1* mice (Number of mice in each group = 7, Student's *t*‐test). (C) Workflow of AAV intratracheal instillation and cigarette smoke exposure. (D) Lung FEV20/FVC, RI, Cchord, Cydn and FRC were performed in 4 groups of mice. Cydn, lung dynamic compliance; Cchord, lung compliance; FRC, functional residual capacity; RI, respiratory index (Number of mice in each group = 7, Student's *t*‐test). (E) Sections of the lung tissues from 4 groups mice were analysed by HE staining (Number of mice in each group = 7, Student's *t*‐test). (F) Sections of the lung tissues from 4 groups mice were analysed by Masson staining (Number of mice in each group = 7, Student's *t*‐test). (G) Sections of the lung tissues from 4 groups mice were analysed by IHC staining of α‐SMA (Number of mice in each group = 7, Student's *t*‐test). (H) Sections of the lung tissues from 4 groups mice were analysed by IHC staining of FN1 (Number of mice in each group = 7, Student's *t*‐test). Data information: Error bars represent means ± SD. *p < .05, **p < .01 and ***p < .05.

To determine if the in vitro findings hold true in vivo, an experiment should be conducted. Nevertheless, it is worth noting that according to NCBI, the lncRNA *HSALR1* is a non‐conserved human lncRNA. Therefore, adeno‐associated virus encoded *HSALR1* (AAV‐*HSALR1*) was constructed and used to express *HSALR1* in mice, which showed the greatest number of genome copies in lung.^31^ Mice were intratracheally administered AAV‐*HSALR1*, then FISH and qRT‐PCR assay showed that adeno‐associated virus successfully causes mice to express *HSALR1* (Figure 7A,B and Figure S11). Human HSP90AB1 exhibits 99.6% amino acid sequence identity to mice HSP90AB1 (Figure S12 and Table S5), which indicated that *HSALR1* can bind to mice HSP90AB1 protein to exert its biological effect.

To explore the role of *HSALR1* in the development of COPD, AAV mice were exposed to smoke for 6 months to establish a model of COPD (Figure 7C). Pulmonary function tests showed that AAV‐*HSALR1* mice displayed poorer lung function in FEV20/FVC, RI, FRC and Cchord. Although RI was not significantly different between Smoke‐NC and Smoke‐*HSALR1*, there was a strong trend towards a difference (Figure 7D). The results of HE stain indicated that compared with NC group, the destruction of alveolar septum was more severe in *HSALR1* group (Figure 7E). Moreover, increased airway thickening and collagen deposition was found in *HSALR1* group (Figure 7F‐H).

**FIGURE 8:**
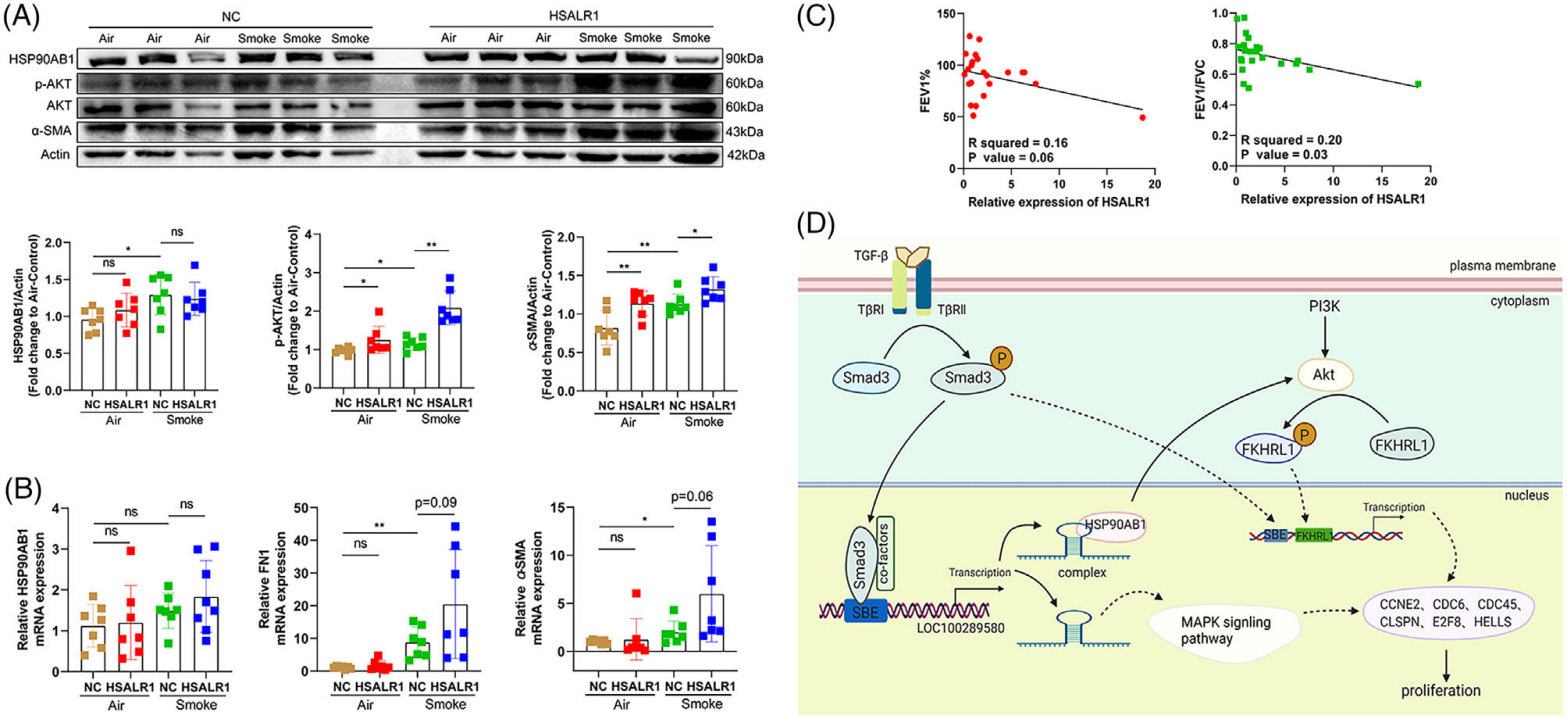
Akt pathway was more activated in *HSALR1* mice. (A) Western blot showing the expression of HSP90AB1, α‐SMA and the activation level of the Akt in 4 groups mice, with Actin as the control. Number of mice in each group = 7, Student's *t*‐test). (B) qRT‐PCR assay showing the expression of *HSP90AB1, α‐SMA* and *FN1* in 4 groups mice, with Actin as the control (Number of mice in each group = 7, Student's *t*‐test). (C) The correlation between FEV1%, FEV1/FVC and the expression of *HSALR1* in human lung tissues. (D) Schematic diagram illustrating the potential mechanism of lncRNA *HSALR1* in TGFβ‐induced AKT activation in HBFs. Data information: Error bars represent means ± SD. *p < .05, **p < .01 and ***p < .05.

Moreover, consistent with the Air group results, results indicated that the phosphorylation of Akt pathway on protein level was increased in *HSALR1* mice (Figure 8A). Notably, while there was a significant increase in HSP90AB1 protein levels observed between Air mice and Smoke mice, no significant change in HSP90AB1 protein levels was observed between NC mice and *HSALR1* mice *HSALR1* (Figure 8A). The protein level of α‐SMA, a significant pathogenic factor of airway remodelling, was found to be significantly elevated in AAV‐*HSALR1* mice, which is consistent with the pathological observations. The results of the qRT‐PCR assay further supported the above results (Figure 8B).

Finally, we found that *HSALR1* expression was significantly negatively correlated with FEV1% and FEV1/FVC, indicating that *HSALR1* might play an important pathogenic role in airway remodelling and COPD (Figure 8C). Altogether, these results suggest that Smad3‐mediated *HSALR1* regulates proliferation of HBFs via the HSP90AB1/Akt axis, and this may contribute to the progression of COPD (Figure 8D).

## 3 DISCUSSION

To date, given that the mechanism of COPD has not been fully elucidated, there are no elaborate treatment strategies for the disease. It is worth noting that COPD has heterogeneous molecular and clinical presentations, which makes it difficult to understand disease aetiology and define robust therapeutic strategies.^32^ In addition, the progression of COPD is very slow and may even take decades, thereby leading to accumulative subtle molecular/cell function and structure alterations. LncRNA, a new category of regulator molecules, has recently emerged as a fine‐tuning system of gene regulation.^14^

This study identified lncRNAs involved in the central process of COPD aetiology and airway remodelling. It is well known that COPD is characterized by the thickening of the bronchial wall caused by interstitial cell proliferation and collagen deposition, where TGF‐β1 plays a key role.^33^ Notably, a TGF‐β1 overexpression activating protein targeting strategy for COPD is currently ongoing.^34^

Unlike the relatively simple Smad‐dependent signalling, the Smad‐independent signalling pathway of TGF‐β1 is very complex and accounts for diverse functions of TGF‐β1. The non‐classical diversity of TGF‐β signalling is primarily influenced by the ‘cross‐talks’ with other signalling pathway adaptors that strengthen, weaken, or modify downstream cellular responses.^34^ It has been previously reported that the Hsp90/Cdc37 chaperone system interacts with and supports 60% of the human kinome, with ‘clients’ present in all kinase families.^35^ Accumulating evidence suggests that HSP90AB1 preferentially binds to RNA. For instance, Cui et al.^36^ found that lncRNA *AC245100.4* promotes proliferation of prostate cancer cells by binding with HSP90. In addition, Zhou et al.^37^ demonstrated that lncRNA PRAL enhances HCC growth by facilitating the combination of HSP90AB1 and p53. A previous study reported that Akt, a non‐classical signal kinase in the TGF‐β1 pathway, is HSP90AB1 dependent and its active form (p‐Akt) is stabilized by forming an intracellular complex with HSP90AB1.^26^ This study found that some lncRNAs were highly associated with TGF‐β in lung tissues. The application of various bioinformatics tools and experimental verifications has identified a promising functional lncRNA, *HSALR1*. Herein, results showed that *HSALR1* is regulated by the TGF‐β/Smad3 signalling pathway, and it can directly bind to HSP90AB1 protein. Although *HSALR1* did not influence the expression of HSP90AB1, it acted as a protein scaffold and maintained the stability of the HSP90AB1‐Akt complex, thereby enhancing activation of the Akt pathway. RNA pulldown assay showed that both HSP90AB1 and Cdc37 were repeatedly in the pool of *HSALR1*‐binding proteins, indicating a relatively high binding affinity. However, only HSP90AB1 was positive in the RIP experiment, implying that it binds to the lncRNA. Overexpression of *HSALR1* enhanced the p‐Akt level and the binding of p‐Akt to HSP90AB1, suggesting that this binding positively affects the pathway. The HSP90‐RNA binding in the cells may directly bridge two pathways via various kinases. The outcome should produce a stronger or quick response in the TGF‐β1‐treated HBFs. Notably, the finding that *HSALR1* is an HSP90AB1‐binding RNA enhances understanding of the TGF‐β’s non‐classical pathway in lung fibroblasts. Furthermore, these results suggest that *HSALR1* promotes activation of the MAPK pathway, but the process might not be through HSP90AB1. Although knockdown of HSP90AB1 suppressed the activity of the MAPK pathway, HSP90AB1 did not bind to MAPK and p‐MAPK directly. Of note, the relationship between HSP90AB1 and COPD has not been reported previously.^38^ Therefore, further studies should be conducted to investigate the regulatory mechanism of HSP90AB1 on the MAPK pathway and its exact functions in COPD disease progression.

Previous studies have proven that the PI3K/AKT and MAPK signalling pathways are two vital pathways downstream of the cell cycle and cell proliferation.^39^, ^40^ Moreover, some studies have shown that these two pathways are implicated in COPD pathogenesis.^40^ Herein, results revealed that *HSALR1* could positively regulate proliferation of HBFs. It was also found that knockdown of the upstream transcription Smad3 and the binding protein HSP90AB1 could partially rescue the effect of cell proliferation by *HSALR1* overexpression. These results suggest that *HSALR1* promotes proliferation of HBFs via the Akt and MAPK pathway.

*HSALR1* is a human‐specific lncRNA, which is not expressed in mice. Thus, we expressed *HSALR1* in mice via intratracheal injection of AAV‐*HSALR1*. Consistent with HBFs analysis results, we observed that *HSALR1* mice spontaneously developed COPD‐like pathological changes including small airway remodelling and collagen deposition. In the context of prolonged exposure to cigarette smoke, COPD progressed more rapidly in *HSALR1* group. HSP90AB1 is a highly conserved protein between human and mice species, and increased phosphorylation of Akt pathway was observed in *HSALR1* mice. According to the results of HBFs in vitro, we concluded that the *HSALR1*/HSP90AB1/Akt axis play a role in promoting COPD progression in mice. Interestingly, the increased protein level of HSP90AB1 was detected in smoke group, but there was no significant difference of HSP90AB1 between AAV‐NC and AAV‐*HSALR1* groups. This was consistent with the results in HBFs, which further explained HSLAR1 acts as a scaffold for HSP90AB1 protein. The certainly is that *HSALR1* might amplify the effect of increasing phosphorylation of Akt in vivo by promoting the protein level of *HSALR1*. However, RNA levels of FN1 and SMA were not significantly different between the AAV‐NC and AAV‐*HSALR1* in air, we speculate that it is due to normal negative feedback regulation, and this negative feedback was destroyed under smoke exposure.

In summary, our results demonstrate that TGF‐β1 can induce the transcription of lncRNA *HSALR1* in fibroblasts via the Smad3 transcription factor. This lncRNA binds to HSP90AB1 and Akt complex component, and enhances the activity of the TGF‐β1 smad3‐independent pathway (Figure 8), ultimately enhancing proliferation of HBF cells and collagen accumulation, the main characteristics of COPD. Expressed *HSALR1* in mice aggravated the progression of COPD in vivo, which were compatible with the results in vitro. Despite the findings, this study had some limitations. For instance, expressing *HSALR1* in mice using an AAV‐based gene delivery vector may not fully reflect its function in humans, and this approach did not account for the potential locus effect of *HSALR1* itself, *HSALR1HSALR1HSALR1*. Otherwise, we investigated the pathogenic mechanism of *HSALR1* in this study, it needs to be validated as a potential biomarker or prognostic indicator of COPD patients in further study. Overall, the findings described here suggest that *HSALR1* is a promising molecular target of COPD therapy.

## 4 MATERIALS AND METHODS

### 4.1 Clinical specimens

This study enrolled 23 patients (13 COPD and 10 non‐COPD patients) admitted in the First Affiliated Hospital of Guangzhou Medical University as previously described^17^ and obtained their lung specimens (Table S2). The patients underwent lung cancer resection or lung volume reduction surgery. The Research Ethics Committee of the First Affiliated Hospital of Guangzhou Medical University (No. 2013−38) approved the study. Signed informed consent was obtained from all patients prior to the study.

### 4.2 Animals and COPD modelling

Twenty‐eight C57/BL6 male mice, 5−6 weeks old, weighing 20–24 g, were obtained from Gempharmatech (Jiangsu, China). Adeno‐associated virus encoding *HSALR1* (AAV‐*HSALR1*) and AAV‐empty vector (AAV‐NC) were purchased from PackGene Biotech (Guangzhou, China). Then, every mouse was infected with a dose of AAV (10^11^ genomic copies) by intratracheal instillation under anaesthesia. Mice were rested 2 weeks after infection to allow for COPD modelling.

Chronic CS‐induced COPD mouse model was established according to protocols described in our previous study.^41^ In summary, 8‐week‐old infected mice were subjected to cigarette smoke exposure. The exposure was conducted 6 days a week, with twice daily sessions lasting 2 h each. The mice were collected after being exposed to cigarette smoke or air for a total of 6 months.

### 4.3 Lung function

Mouse lung function was performed by using an invasive mouse pulmonary function system as described previously.^42^ Briefly, after system calibration, mice were anesthetized via intraperitoneal injection of sodium pentobarbital (50 mg/kg), tracheostomized, and placed in the DSI Buxco Pulmonary Function Test (PFT) (Buxco Research Systems, USA). Chord compliance (Cchord), dynamic compliance (Cdyn), functional residual capacity (FRC), airway resistance (RI) and forced expiratory volume in 20 ms (FEV20) were measured.

### 4.4 Cell culture

Primary HBFs and fibroblast medium (FM) were obtained from ScienCell (USA). HBFs at passages 4−8 were cultured in fibroblast medium containing 2% foetal bovine serum (FBS; ScienCell) supplemented with 1% penicillin‐streptomycin (P/S; ScienCell) and 1% fibroblast growth supplement (ScienCell). Human lung fibroblast 1 (HFL1, ATCC CCL‐153) cells, F‐12K (Kaighn's modification of Ham's F‐12 medium, ATCC 30−2004), BEAS‐2B (ATCC CRL‐9609) cells, A549 (ATCC CCL‐185) and RPMI‐1640 medium (ATCC 30−2001) were obtained from ATCC (Manassas, USA). HFL1 cells were cultured in F‐12K medium containing 10% FBS (Gibco, USA) and 1% P/S (Invitrogen, USA), whereas BEAS‐2B and A549 cells were cultured in RPMI‐1640 medium containing 10% FBS and 1% P/S. The above primary cells or cell lines were all incubated at 37°C under a humidified atmosphere containing 5% CO_2_.

### 4.5 RNA extraction, cDNA synthesis and qRT‐qPCR

Total cellular and tissue RNA were extracted using Trizol reagent (Invitrogen) as previously described.^43^ A PrimeScript™ RT reagent kit with gDNA Eraser (Takara, Japan) was then used to synthesize cDNA from 1000 ng total RNA in accordance with the manufacturer's protocol. qRT‐PCR was conducted using TB Green Premix Ex Taq II (Tli RNaseH Plus, Takara) and analysed on a CFX connect real‐time PCR detection system (Bio‐Rad, USA). The relative expression levels of the unigenes were calculated using the 2^−∆∆^ CT method, with GAPDH as the internal control. All specific primers used in this study are listed in Table S4.

### 4.6 Gene knockdown and overexpression

RiboBio Technology Co., Ltd. (China) supplied the siRNAs of *HSALR1*, ENST00000587755, ENST00000567968, ENST00000499732, Smad3 and HSP90AB1. ShRNA lentivirus of ENST0000044040 (two target shRNA lentiviruses, sh‐*HSALR1*‐1 and sh‐*HSALR1*‐2) and a negative control shRNA lentivirus (sh‐NC), lentivirus overexpression vectors of *HSALR1* (oe‐*HSALR1*) and control (oe‐NC) were purchased from Shanghai Ji Kai Gene Technology Co., Ltd. All sequence information is shown in Table S4.

For subsequent in vitro experiments, HBFs, HFL1, A549 and BEAS‐2B cells were transfected with siRNA using Lipofectamine 3000 (Thermo Fisher Scientific) following the manufacturer's instructions. HBFs cells were also infected with lentivirus containing *HSALR1* overexpressing vector or *HSALR1* shRNA vector according to the manufacturer's instructions.

For co‐transfection, HBFs were transfected with siRNA of Smad3 and HSP90AB1 for 48 h after a 24‐h infection with lentivirus containing *HSALR1* overexpression vector.

### 4.7 Cell counting kit‐8 assay

CCK‐8 assay (Dojindo, Japan) was performed to determine cell viability after transfection or stimulation according to the manufacturer's instructions. A spectrophotometer was used to measure absorbance at 450 nm.

### 4.8 EdU assay

The HBFs were seeded into 96‐well plates and transfected or infected with siRNA or lentivirus. The EdU incorporation assay was then performed using a Cell‐LightTM EdU Apollo567 in vitro Kit (RiboBio, China) following the manufacturer's instructions. Finally, Leica DMi8 was used to visualize the relative proliferation rate of cells in each group.

### 4.9 The dual‐luciferase assay

This study constructed *HSALR1* promoter wild‐type (GV354‐wt) and site‐mutant (GV354‐mut‐s1, GV354‐mut‐s2 and GV354‐mut‐s1/2) dual‐luciferase reporter vector. The HBFs were seeded into 96‐well plates and then transfected or infected with dual‐luciferase reporter vector. A Dual‐Luciferase® Reporter 1000 Assay System was used to detect the relative luciferase activity.

### 4.10 RNA fluorescence in situ hybridization and protein‐RNA double labelling (IF/FISH) assay

The FISH probes of *HSALR1* were purchased from RiboBio Technology Co. Ltd. (China). FISH assay was performed as previously described.^18^ The FISH kit (RiboBio) was used to recognize the cellular localization and subcellular localization of *HSALR1* following the manufacturer's protocol. Finally, Leica DM6 M was used to visualize the relative fluorescence of cells in each group.

The IF/FISH assay was performed as previously described.^44^ The FISH assay was carried out following the manufacturer's protocol, and subsequently, the lung tissue sections were incubated with antibodies against human FN1 (1:100, sc‐271098, Santa Cruz) and Vimentin (1:100, ab8978, Abcam) for 2 h at 37°C, followed by incubation with Alexa Fluor 488 goat anti‐rabbit IgG (H + L) (1:500, Invitrogen) for 40 min. Images were captured using the Leica DM6 M.

### 4.11 RNA pulldown assay and mass spectrum

RNA pulldown assay and mass spectrum were performed as previously described.^18^ Briefly, ENST0000440406 overexpression vector was used as a template to perform PCR in vitro amplification of the sense and antisense strands of *HSALR1* using primers with a T7 promoter. A TranscriptAid T7 High Yield Transcription Kit (Thermo) was used to prepare biotinylated *HSALR1*, and then the Magnetic RNA‐Protein Pull‐Down Kit (Pierce 20164, USA) was used to perform the RNA pulldown assay following the manufacturer's instructions. Next, Western blot, silver staining and mass spectrometry steps were performed as previously described.^18^

### 4.12 Western blot

Western blot analysis was performed as previously described.^45^ Briefly, cells or/and tissues were lysed in RIPA lysis buffer (89901, Thermo Fisher Scientific) containing a protease inhibitor cocktail (78430, Thermo Fisher Scientific) at 4°C for 15 min. The protein extracts were resolved through 10% SDS‐polyacrylamide gel electrophoresis and transferred to polyvinylidene difluoride membranes (BioRad). After blocking, membranes were incubated with antibodies against human SMAD3 (C67H9, CST, USA), HSP90AB1 (5087S, CST, USA), AKT (4691S, CST, USA), p‐AKT (ab81283, Abcam, UK), p‐p65 (ab76302, Abcam, UK), p65 (ab32536, Abcam, UK), MAPK (Erk1/2) (9107S, CST, USA), p‐MAPK (Erk1/2) (4370S, CST, USA), NLRP3 (ab263899, Abcam, UK), CDC37 (10218‐1‐AP, Proteintech, China) and GAPDH (10494‐1‐AP, Proteintech, China) overnight at 4°C. On the next day, membranes were washed and then incubated with a peroxidase‐conjugated secondary antibody (Proteintech). Finally, cells were visualized (chemiluminescence) using Amersham Imager 680 (Thermo Fisher Scientific).

### 4.13 Haematoxylin–eosin, Masson and immunohistochemistry staining

Haematoxylin–eosin (HE) and immunohistochemistry (IHC) staining were performed as described in our previous study.^41^ In brief, tissues were paraffin‐embedded, dewaxed, rehydrated and stained with HE and Masson. For IHC, tissue sections of lungs from mice were incubated with antibody against mouse α‐SMA (19245S, CST, USA), followed by incubation with biotin‐conjugated Affinipure Goat Anti‐Rabbit IgG (H + L) (SA00004‐2, Proteintech, China). Further, the images were captured using a Digital Pathology Scanner (Aperio CS2, Leica). The mean linear intercept (MLI) was then used to estimate the average diameter of a single alveolus using the formula:

MLI = Total length/Alveolar septal number.

Airway wall thickness was assessed by segmental airway wall area percentage (Segmental WA%  =  [Outer bronchus area − Airway luminal area]/Outer bronchus area).

ImageJ software was used to measure airway area and Masson positive area and to calculate as percentage of stained area to total airway wall area (collagen area/airway area). The same statistical methods were used for IHC of α‐SMA (α‐SMA area/airway area) and FN1 (FN1 area/airway area).

### 4.14 RNA immunoprecipitation

RIP assay was conducted in accordance with a previously described protocol.^18^ The RIP assay was performed using a Magna RIP RNA‐Binding Protein Immunoprecipitation Kit (Millipore Sigma 17−700, USA) with antibodies against IgG (Millipore Sigma 17−700, USA), HSP90AB1 (5087S, CST, USA) and CDC37 (10218‐1‐AP, Proteintech, China) following the manufacturer's instructions.

### 4.15 Chromatin immunoprecipitation

ChIP assay was conducted as previously described.^18^ The ChIP assay was performed using a SimpleChIP Enzymatic Chromatin IP Kit (9004S, CST, USA) with antibodies against IgG (9004S, USA) and SMAD3 (C67H9, CST, USA) following the manufacturer's instructions. The final ChIP DNA was used as a template in qPCR with the primers listed in Table S4.

### 4.16 Co‐immunoprecipitation

The Pierce Classic Magnetic IP/Co‐IP Kit (Thermo Fischer Scientific, 88804, USA) was used to conduct the Co‐IP assay with antibodies against IgG (Millipore Sigma 17−700, USA), HSP90AB1 (5087S, CST, USA), AKT (4691S, CST, USA), p‐AKT (ab81283, Abcam, UK) and CDC37 (10218‐1‐AP, Proteintech, China) following the manufacturer's instructions. Briefly, HBF cells infected with the lentivirus were harvested and lysed in lysis buffer on ice. The cell lysates were then incubated overnight at 4°C with Co‐IP buffer containing magnetic beads conjugated with the indicated antibody or anti‐IgG. Finally, the beads were washed, the bound protein‐DNA complexes were eluted, and then Western blot analysis was performed to determine the denatured proteins.

### 4.17 Cell cycle

Flow cytometric analysis (FACS) was used to evaluate the cell cycle via propidium iodide (PI) staining (550825, BD, USA) following the manufacturer's instructions. Notably, the HBFs were stained with PI after transfection or infection with siRNA or lentiviruses. Briefly, the HBFs were collected via trypsin digestion, fixed with chilled 70% ethanol (added dropwise) and then incubated overnight at 4°C. On the next day, cells were washed with PBS, and then incubated with PI/RNase staining buffer (550825, BD) in the dark at room temperature for 30 min. The cells were then subjected to FACS analysis on a FACScan flow cytometer (BD PharMingen). ModFit software was used for data analysis.

### 4.18 RNA‐seq and bioinformatics

Sequencing of associated RNA samples was performed at BGI (China) using the BGISEQ‐500 system. The RNA‐seq data were aligned to the Ensembl v86 transcript annotations. ‘Limma’ package in R software was used to identify the differentially expressed genes (DEGs). The DE mRNAs were subjected to gene ontology (GO) functional enrichment (http://geneontology.org/)^46^ and Kyoto Encyclopaedia of Genes and Genomes (KEGG) pathway analysis (http://www.kegg.jp/).^47^ Next, phyloCSF was used to calculate the coding potential of *HSALR1*, which was visualized in the UCSC genome browser. The potential binding sites of Smad3 on the promoter of *HSALR1* were predicted using JASPAR (http://jaspar.genereg.net/). PPI networks of associated genes were constructed using the STRING database (http://string‐db.org)^48^ and visualized using Cytoscape (v.3.6.1).^49^ Finally, the top 10 hub genes were obtained using Cytohubba.

### 4.19 Statistical analyses

All statistical analyses were performed using GraphPad Prism v8.0.1. A two‐tailed paired Student's *t*‐test and one‐way analysis of variance (ANOVA) test were used to determine the significance between means. The *p‐*values were represented as follows: ns (not significant), *p < .05, **p < .01 and ***p < .001.

## CONFLICT OF INTEREST STATEMENT

The authors declare no conflicts of interest.

## Supporting information

Supporting Information

## Data Availability

Raw data associated with the main and supplementary figures are available upon reasonable request. The data are not publicly available due to privacy or ethical restrictions.
