# Supplementary material for: Smad3‐mediated lncRNA HSALR1 enhances the non‐classic signalling pathway of TGF‐β1 in human bronchial fibroblasts by binding to HSP90AB1
Source: Clin Transl Med. 2023 Jun 14;13(6):e1292. doi: 10.1002/ctm2.1292 (PMC10267427; doi:10.1002/ctm2.1292)
Supplement: Supplementary file 1 — Supporting Information [file CTM2-13-e1292-s001.docx]

Supplementary Materials


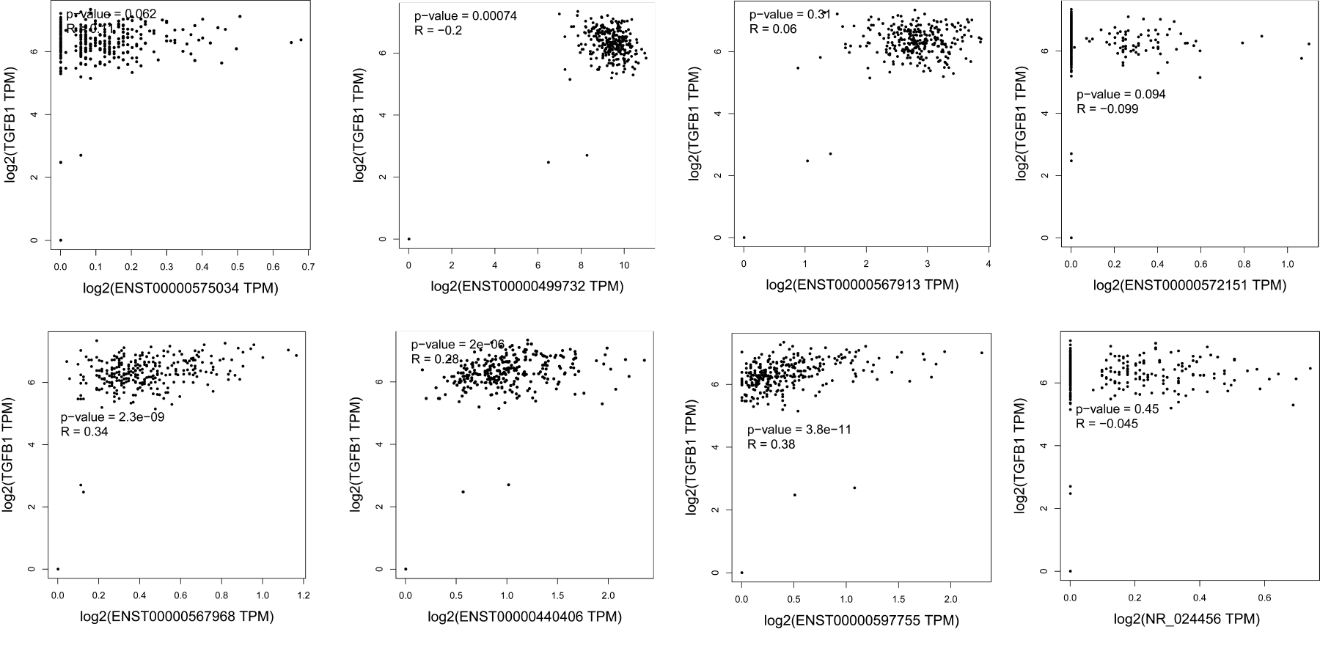


**Fig.S1**

Correlation analysis between the expression of top eight most correlated lncRNAs and TGF-β expression in lung tissues based on GEPIA database.


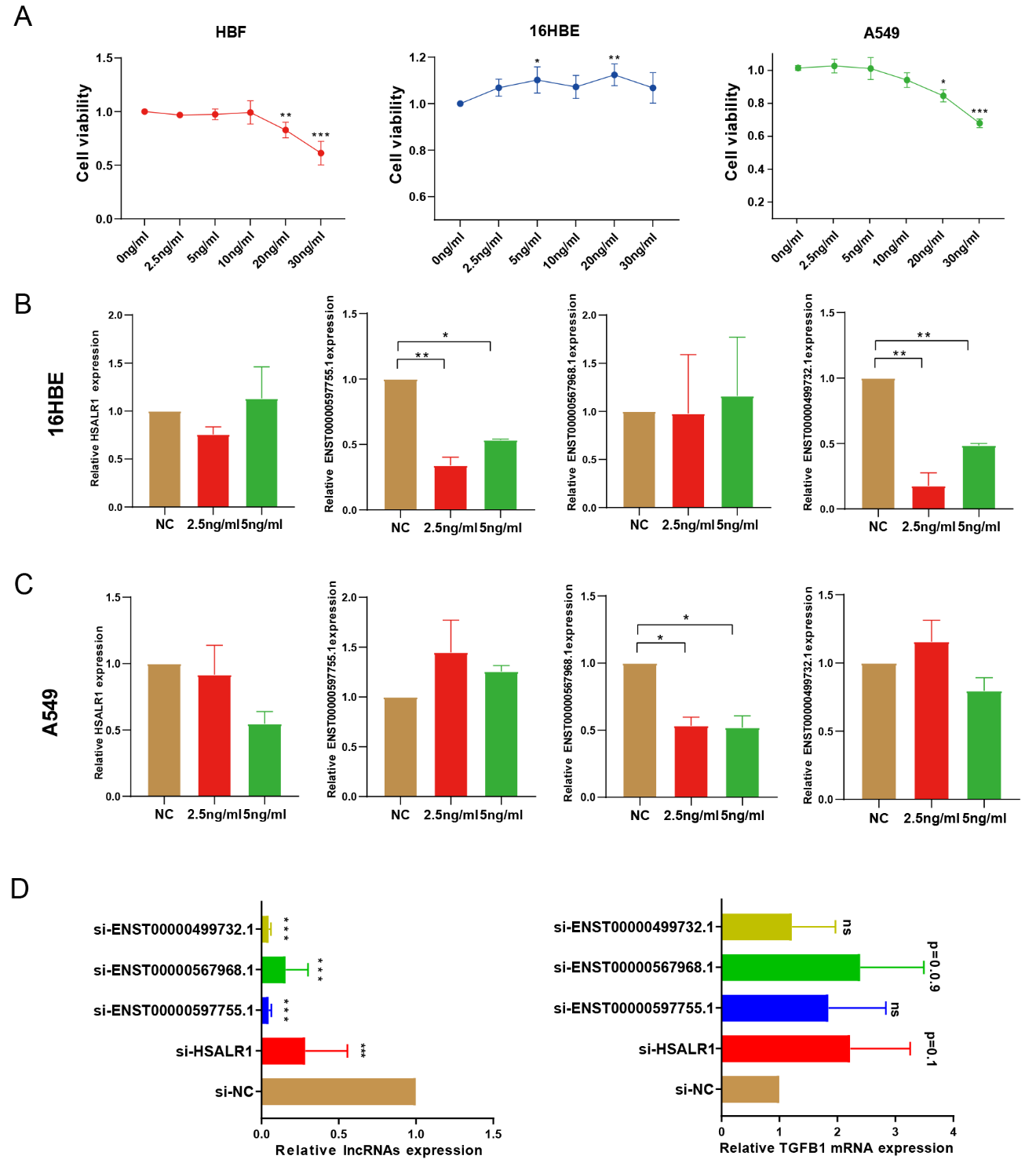


**Fig.S2**

**A** CCK-8 assay was performed after TGF-β (0ng/ml, 2.5ng/ml, 5ng/ml, 10ng/ml, 20ng/ml, 30ng/ml) stimulated for 48h in HBF cells, 16HBE and A549. (one-way ANOVA, n=3 biological replicates).

**B-C** qRT-PCR analysis of the expression of *HSALR1*, *ENST00000597755, ENST00000567968* and *ENST00000499732* (*HSALR1*, *lnc-10, lnc-12* and lnc-15 in graphs) after TGF-β stimulation (0ng/ml, 2.5ng/ml, 5ng/ml) in 16HBE **(B)** and A549 **(C)** for 48h. (n=3 biological replicates, one-way ANOVA)

**D** RT-PCR analysis of the expression of *HSALR1*, *ENST00000597755.1, ENST00000567968.1* and *ENST00000499732.1* after knockdown using specific siRNA in HBF cells. (n=3 biological replicates, one-way ANOVA). qRT-PCR analysis of TGF-β expression after *HSALR1*, *ENST00000597755.1, ENST00000567968.1* and *ENST00000499732.1* knockdown using siRNA in HBF cells. (n=3 biological replicates, one-way ANOVA)

Data information: Error bars represent means ± SD. **P*< 0.05, ***P*< 0.01 and ****P*< 0.001.


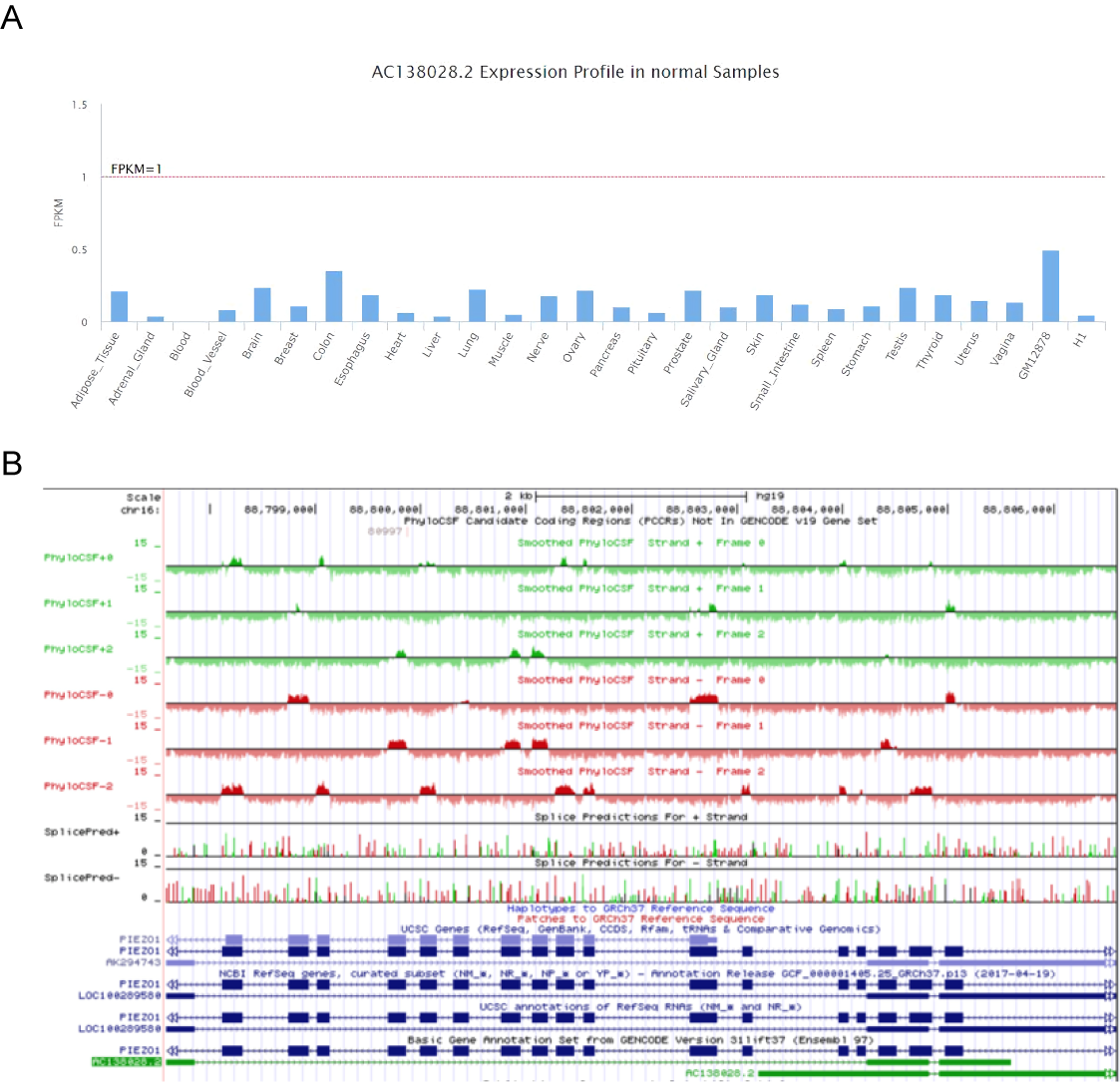


**Fig.S3**

**A** LncRNA *HSALR1* expression in various human tissues in the AnnoLnc2 database.

q

**B** Coding potential of *HSALR1* predicted using PhyloCSF analysis.


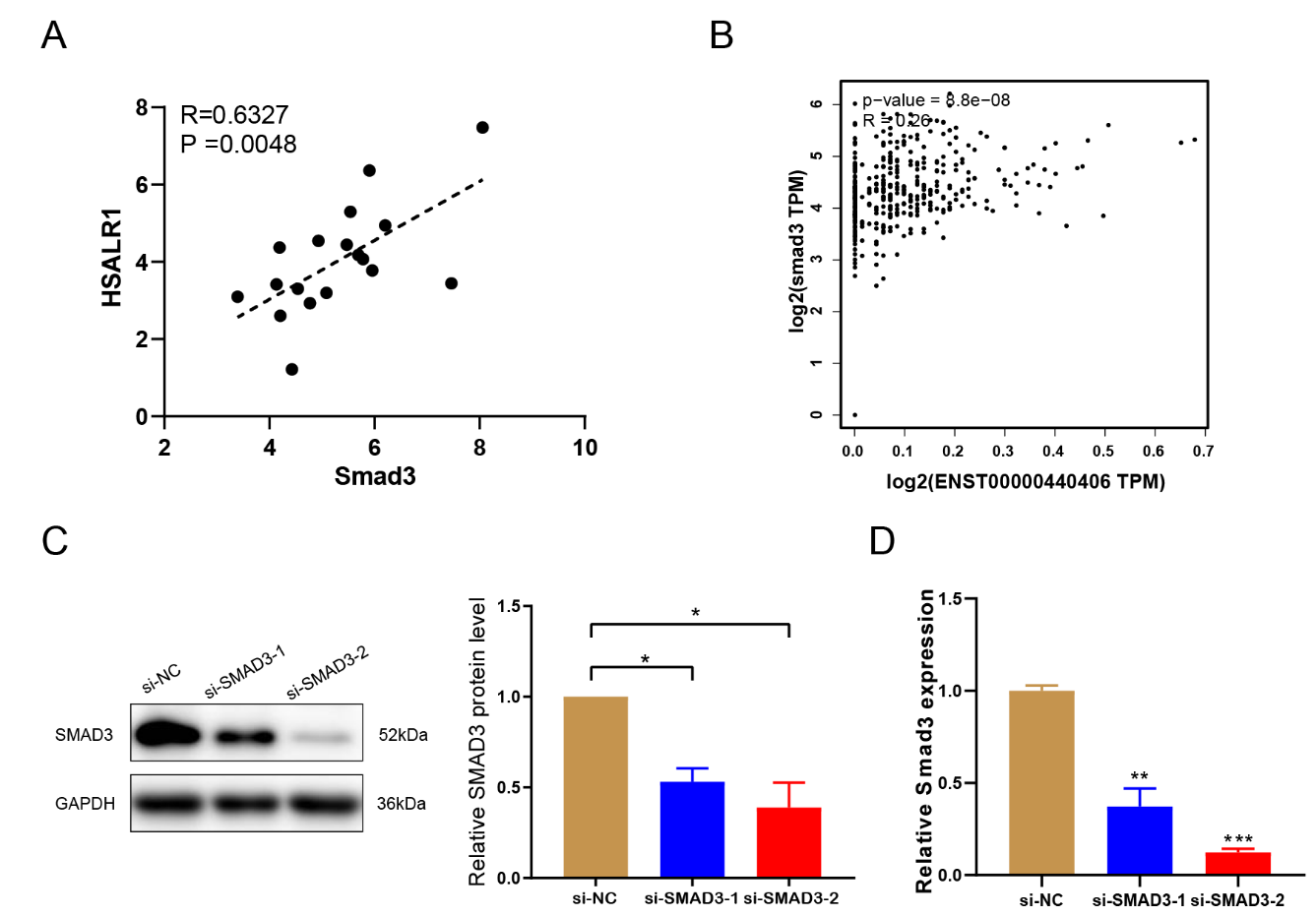


**Fig.S4**

**A** Correlation analysis between *HSALR1* and *Smad3* expressions in RNA-seq results.

**B** Correlation analysis between *HSALR1* and *Smad3* expressions in GEPIA database.

**C-D** Western blot (**C**) and qRT-PCR (**D**) analysis of Smad3 expression after transfection with two siRNAs (siSmad3-1 and siSmad3-2) in HBF cells. (n=3 biological replicates, one-way ANOVA)

Data information: Error bars represent means ± SD. **P*< 0.05.


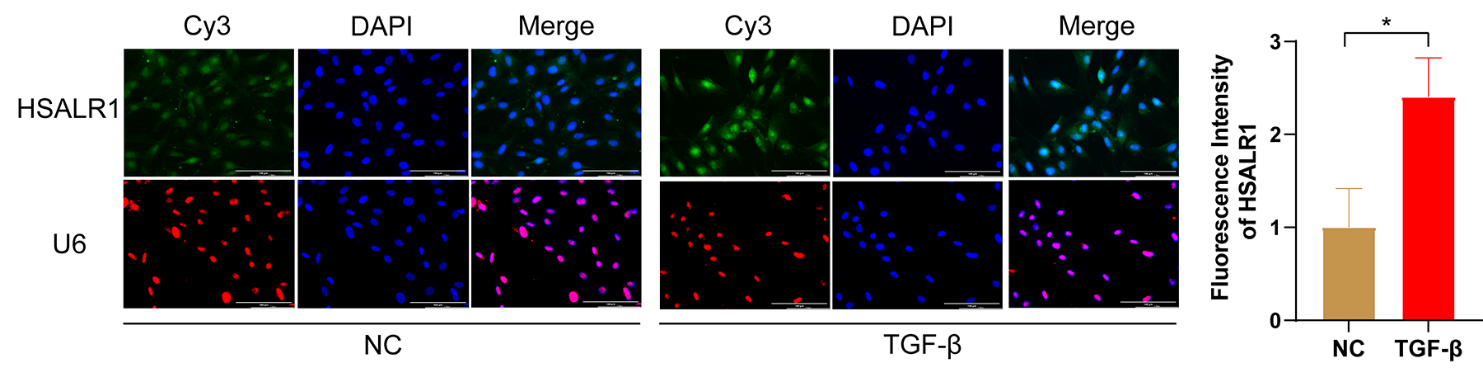


**Fig.S5**

FISH assay showing the subcellular localization of *HSALR1* in HBFs after TGF-β stimulation. U6 was used as a positive control for nuclear localization; Blank was used as the blank control with negative probes. Green, *HSLAR1*; Read, U6; Blue, DAPI. (n=3 biological replicates, Student’s t-test)
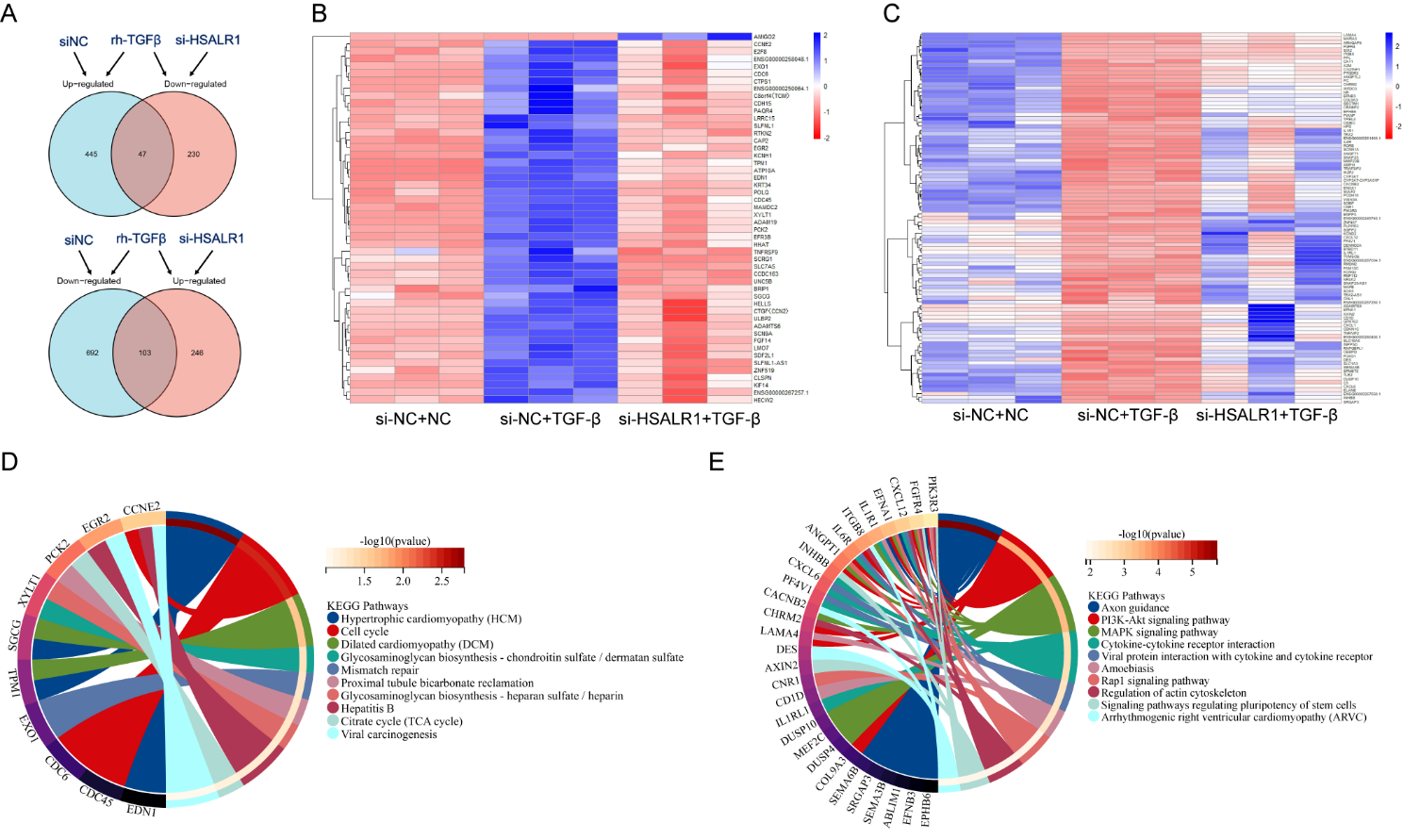


**Fig.S6**

**A.** The intersection of up-regulated genes in si-NC+NC vs si-NC+TGF-β group and down-regulated genes in si-NC+TGF-β vs si-lnc9+TGF-β group, and the down-regulated genes in si-NC+NC vs si-NC+TGF-β group and up-regulated genes in si-NC+TGF-β vs si-*HSALR1*+TGF-β group. (n=3 biological replicates)

**B-C.** The heatmaps representing 47 overlapping genes **(B)** in Figure.S2A or 103 overlapping genes **(C)** in Figure.S2B. (n=3 biological replicates)

**D-E.** Schematic representation of the KEGG analysis 47 overlapping genes **(D)** or 103 overlapping genes **(E)**.


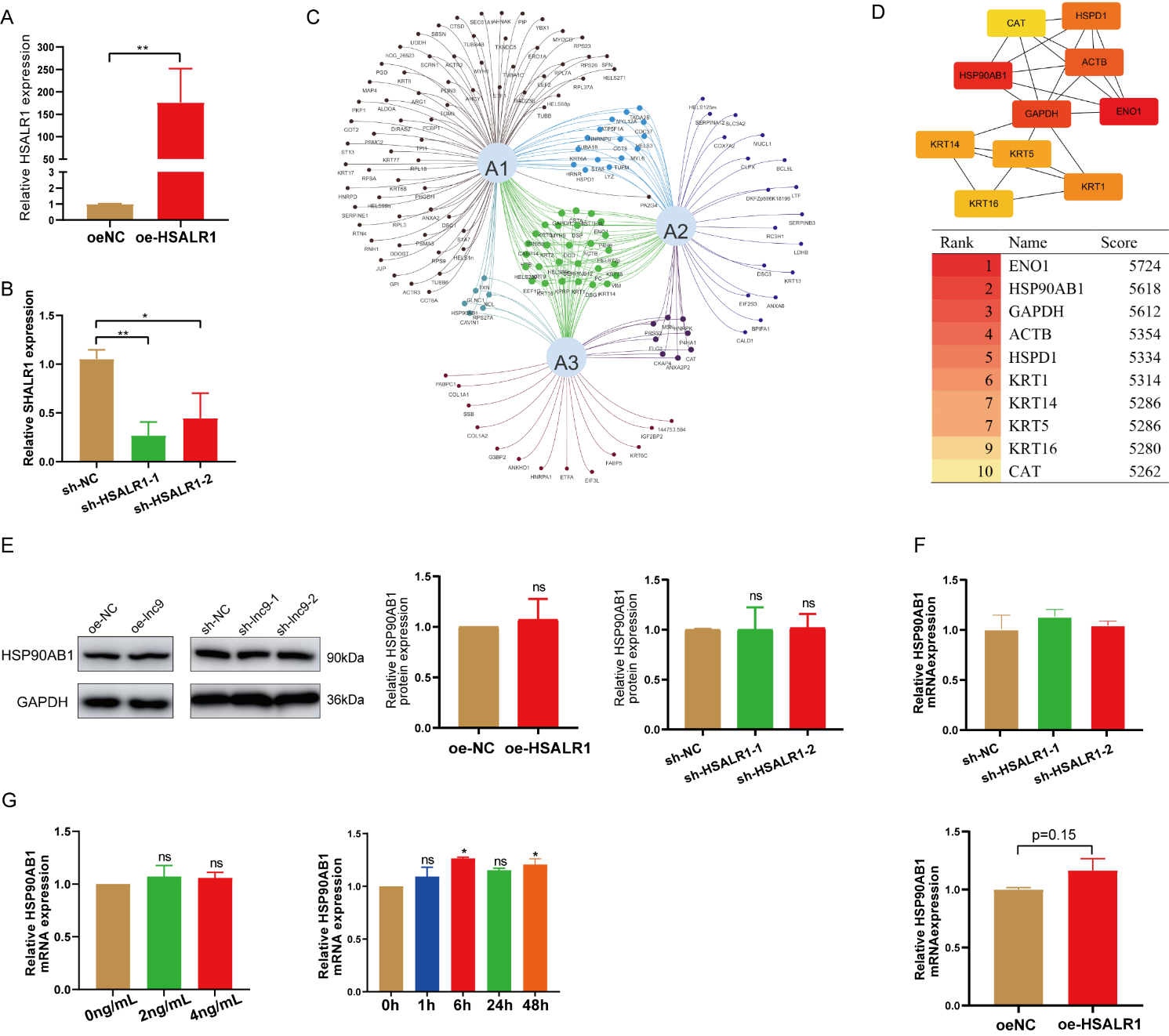


**Fig.S7**

**A-B.** qRT-PCR analysis of *HSALR1* expression after *HSALR1* knockdown using shRNA lentivirus **(A)** or *HSALR1* overexpression using overexpression lentiviral vector **(B)** in HBF cells. (n=3 biological replicates, Student’s t-test)

**C.** Venn diagram showing the overlapping genes among the three independent RNA-pulldown experiments. (n=3 biological replicates)

**D.** Schematic representation of the top 10 genes and their score in the PPI network of the overlapping genes in Fig.5B.

**E-F.** Western blot **(E)** and qRT-PCR **(F)** analysis of *HSP90AB1* expression after infection with *HSALR1* siRNA or overexpression vector in HBF cells. (n=3 biological replicates, Student’s t-test)

**G.** qRT-PCR analysis of *HSP90AB1* expression after TGF-β stimulation with 2ng/mL and 4ng /mL at 6h, and TGF-β stimulation for various time points (0h, 1h, 24h, 24h). (n=3 biological replicates, Student’s t-test)

Data information: Error bars represent means ± SD. **P* < 0.05 and ***P* < 0.01.


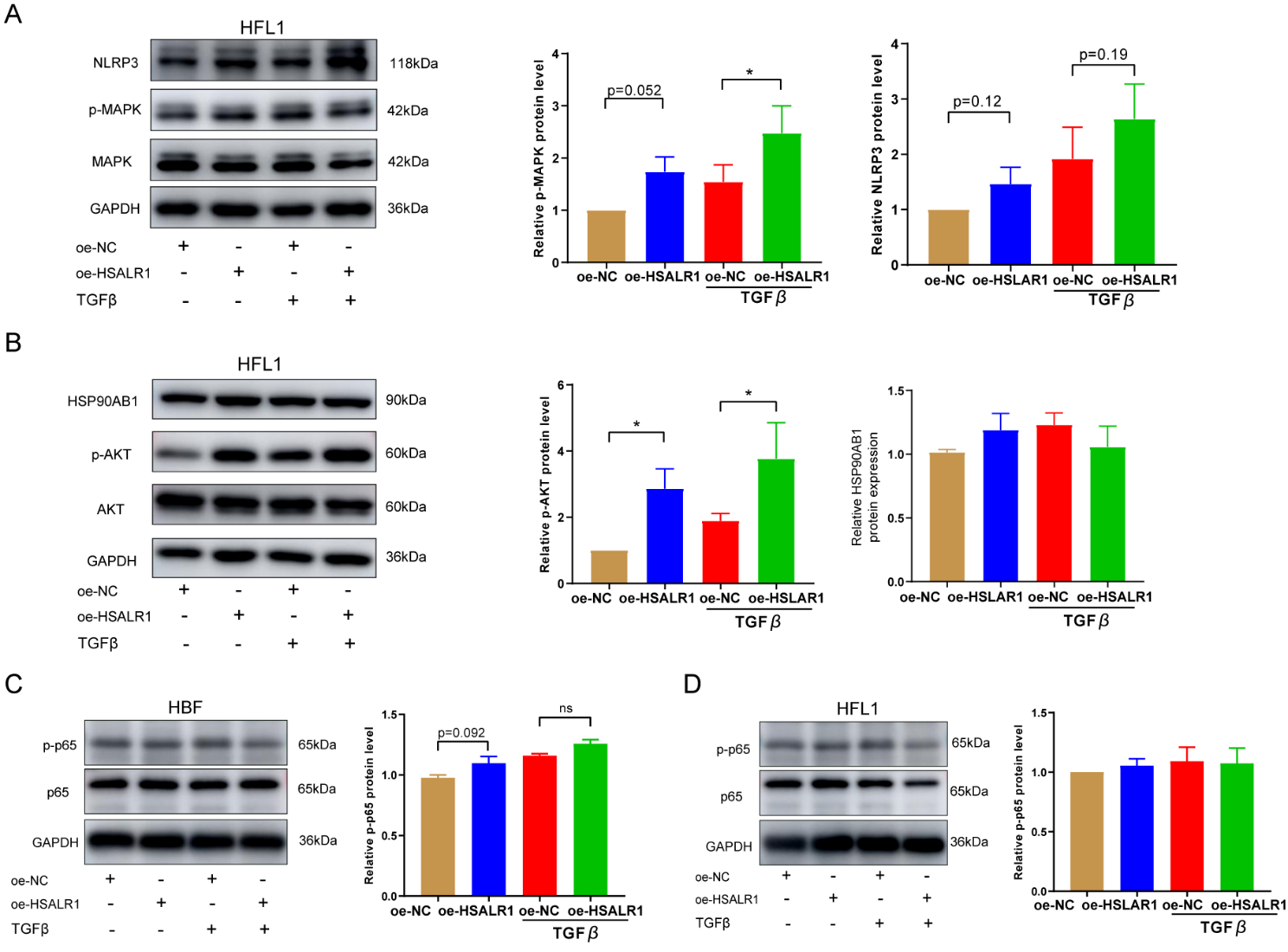


**Fig.S8**

**A.** Western blot showing the protein levels of NLRP3, MAPK, and p-MAPK after transfection with *HSALR1* overexpression vector and stimulation with TGF-β in HFL1 cells. (n=3 biological replicates, one-way ANOVA)

**B.** Western blot showing the protein levels of HSP90AB1, Akt, and p-Akt after transfection with *HSALR1* overexpression vector and stimulation with TGF-β in HFL1 cells. (n=3 biological replicates, one-way ANOVA)

**C-D.** Western blot showing the protein levels of p-p65 and p65 after transfection with *HSALR1* overexpression vector and stimulation with TGF-β in HBF **(C)** and HFL1 **(D)**. (n=3 biological replicates, one-way ANOVA)

Data information: Error bars represent means ± SD. **P* < 0.05.


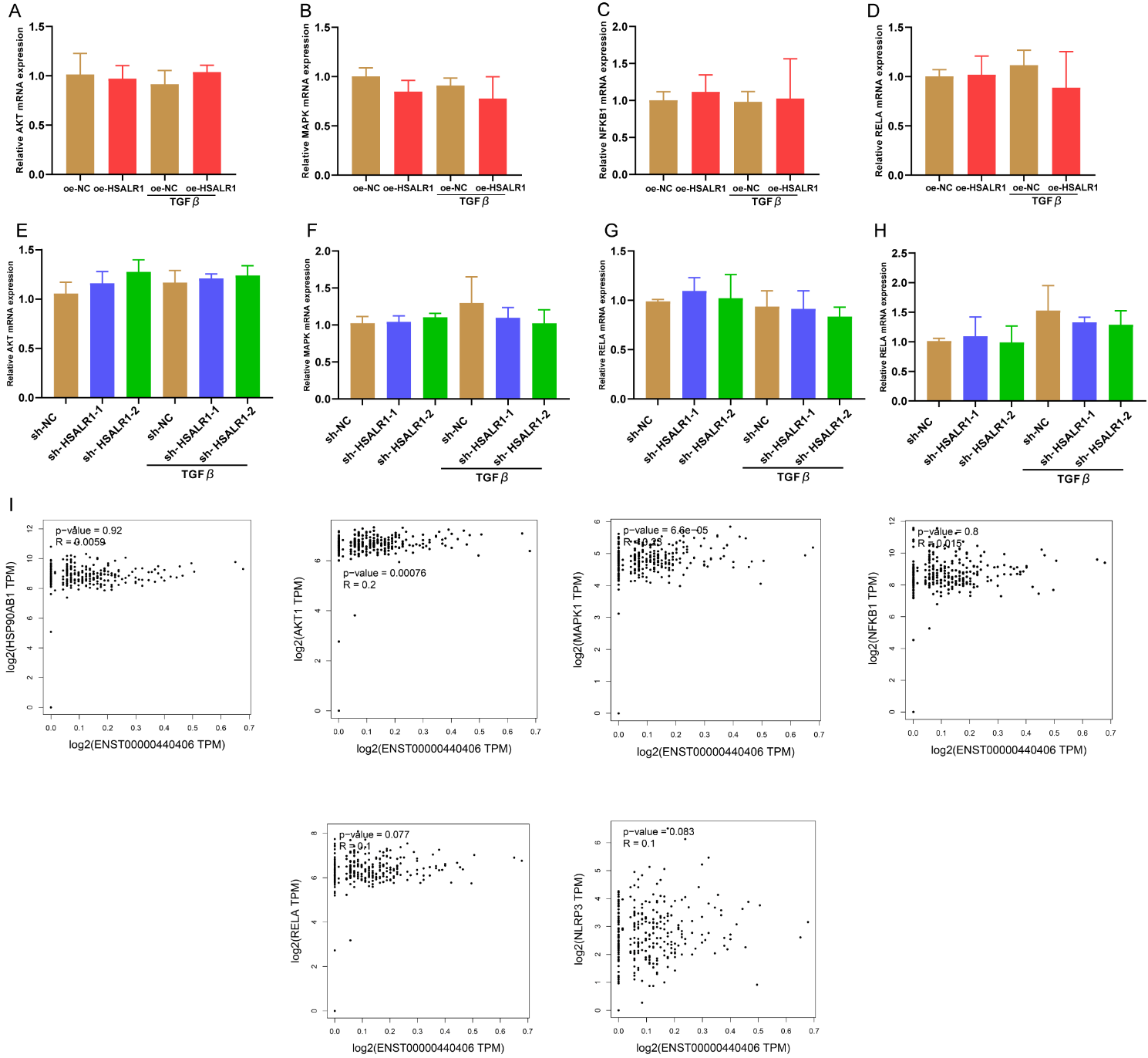


**Fig.S9**

**A-D.** qRT-PCR analysis of *AKT* **(A)**, *MAPK* **(B)**, *NFKB1* **(C),** and *RELA* **(D)** expression after transfection with *HSALR1* overexpression vector in HBF cells. (n=3 biological replicates, one-way ANOVA)

**E-H.** qRT-PCR analysis of *AKT* **(E)**, *MAPK* **(F)**, *NFKB1* **(G),** and *RELA* **(H)** expression after transfection with *HSALR1* shRNA-1 and *HSALR1* shRNA-2 in HBF cells. (n=3 biological replicates, one-way ANOVA)

**I.** Correlation analysis of gene expression between *HSALR1* and *HSP90AB1* in human lung tissues based on the GEPIA database.

**J.** Correlation analysis of gene expression between *HSALR1* and *Akt1, MAPK1, NLRP3, RELA, NFKB1* in human lung tissues based on the GEPIA database.

Data information: Error bars represent means ± SD.


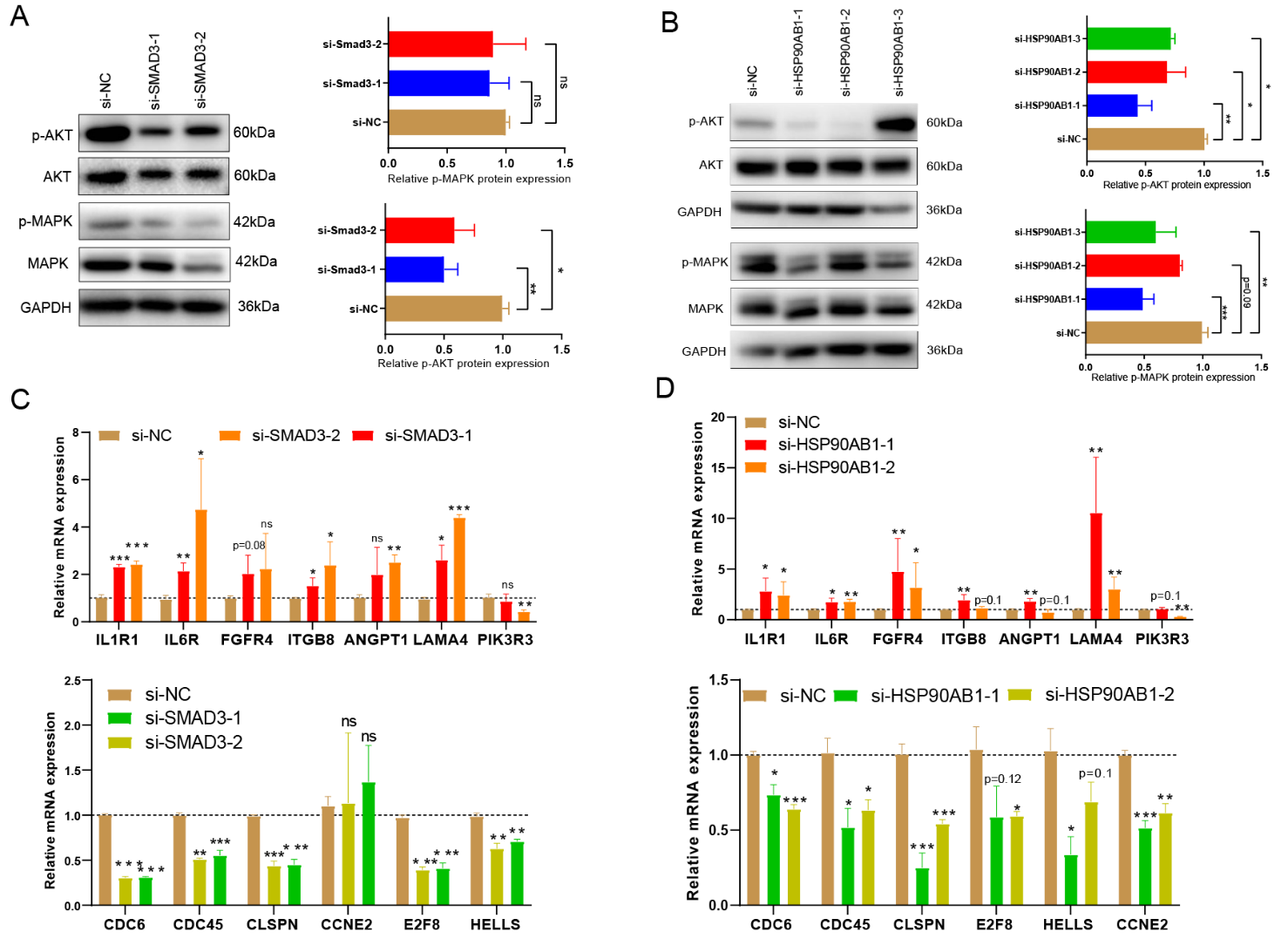


**Fig.S10**

**A** Western blot showing the expression of HSP90AB1, the activation level of the Akt and MAPK signaling pathway in HBFs after Smad3 knockdown using si-Smad3-1 and si-Smad3-2, with GAPDH as the control. (n=3 biological replicates, one-way ANOVA)

**B** Western blot showing the expression of HSP90AB1, the activation level of the Akt and MAPK signaling pathway in HBFs after knockdown of HSP90AB1 using si-HSP90AB1-1, si-HSP90AB1-2 and si-HSP90AB3-1, with GAPDH as the control. (n=3 biological replicates, one-way ANOVA)

**C** qRT-PCR analysis of above proliferation-associated genes and cytokine-associated genes after Smad3 knockdown using two siRNA (si-Smad3-1 and si-Smad3-2) in HBF cells. (n=4 biological replicates, Student’s t-test)

**D** qRT-PCR analysis of above proliferation-associated genes and cytokine-associated genes after HSP90AB1 knockdown using siRNA (si-HSP90AB1-1 and si-HSP90AB1-2) in HBF cells. (n=4 biological replicates, Student’s t-test)

Data information: Error bars represent means ± SD. **P* < 0.05, ***P* < 0.01 and ****P* < 0.001.

**Fig.S11**


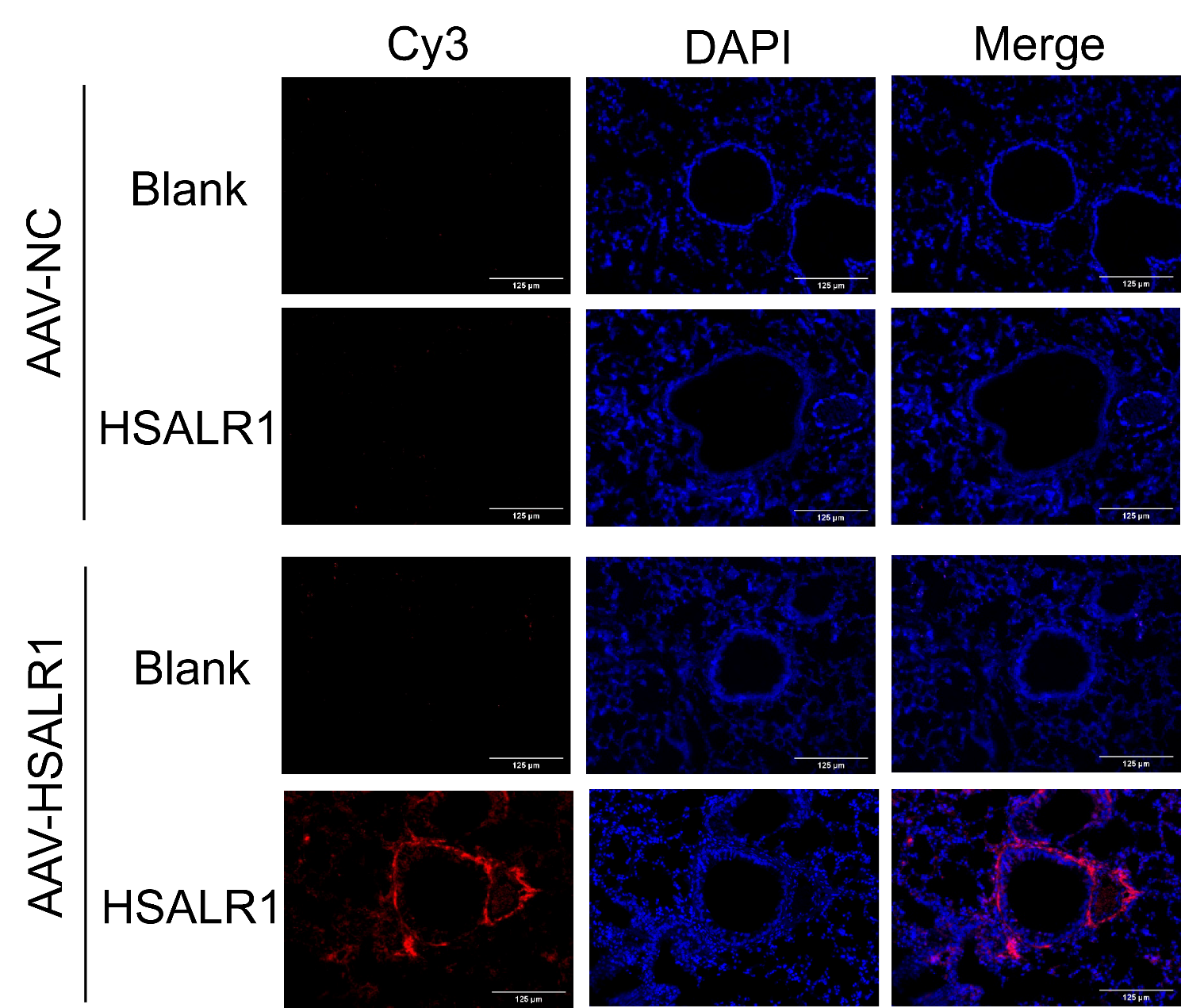


FISH assay showing the localization of *HSALR1* in AAV-NC mice and AAV-*HSALR1* mice; Blank was used as the blank control with negative probes. Red, HSLAR1; Blue, DAPI. (n=3 biological replicates, the number of images observed = 3)

**Fig.S12**


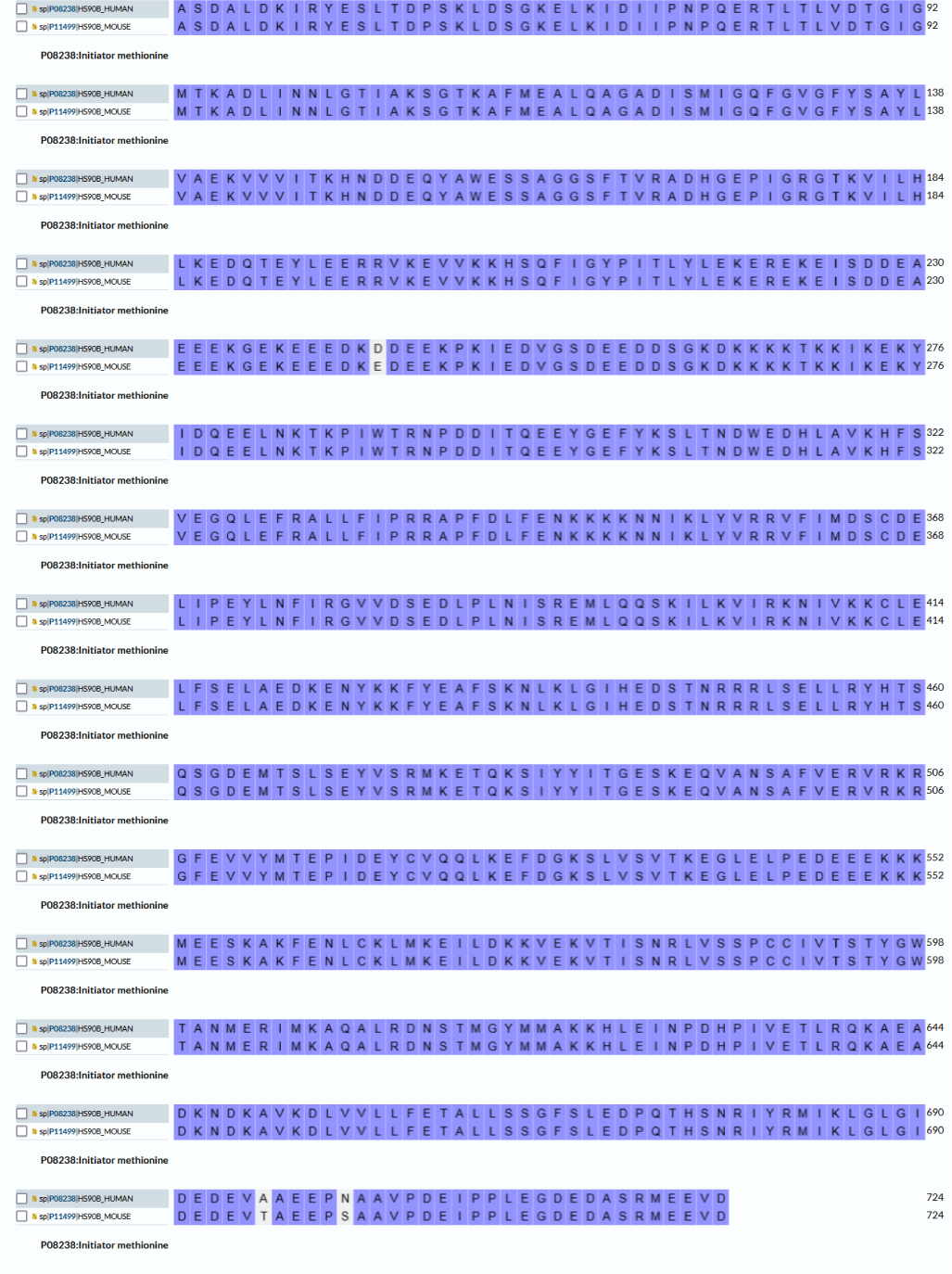


The conservation of protein HSP90AB1 between humans and mice.

**Supplementary Table 1.** **Clinical characteristics of the subjects for sample validation**

| mRNA | lncRNA | Cor | | Zscore | Pvale |
| --- | --- | --- | --- | --- | --- |
| TGFB1 | NR_029376 | 0.87260039 | | 3.608191977 | 3.08E-04 |
| TGFB1 | ENST00000575034.1 | 0.87244415 | | 3.586821296 | 3.35E-04 |
| TGFB1 | NR_037942 | 0.86799478 | | 3.575579972 | 3.49E-04 |
| TGFB1 | NR_037943 | 0.86772223 | | 3.57556566 | 3.49E-04 |
| TGFB1 | ENST00000544868.1 | 0.85570171 | | 3.531243015 | 4.14E-04 |
| TGFB1 | NR_037941 | 0.85222151 | | 3.510538442 | 4.47E-04 |
| TGFB1 | NR_046474 | 0.8508621 | | 3.509635542 | 4.49E-04 |
| TGFB1 | ENST00000440406.2 | 0.85399978 | | 3.50211213 | 4.62E-04 |
| TGFB1 | ENST00000567968.1 | 0.83336929 | | 3.470452148 | 5.20E-04 |
| TGFB1 | NR_027151 | 0.8354999 | | 3.436172869 | 5.90E-04 |
| TGFB1 | ENST00000601801.1 | 0.82225428 | | 3.419499443 | 6.27E-04 |
| TGFB1 | NR_024456 | 0.81873297 | | 3.399845028 | 6.74E-04 |
| TGFB1 | NR_033649 | 0.82732318 | | 3.39941614 | 6.75E-04 |
| TGFB1 | ENST00000572151.1 | 0.81902264 | | 3.3921694 | 6.93E-04 |
| TGFB1 | NR_033650 | 0.82732479 | | 3.386339059 | 7.08E-04 |
| TGFB1 | NR_027473 | 0.82203164 | | 3.379424878 | 7.26E-04 |
| TGFB1 | ENST00000439298.1 | 0.81758371 | | 3.365469308 | 7.64E-04 |
| TGFB1 | NR_037162 | 0.80825035 | | 3.332748063 | 8.60E-04 |
| TGFB1 | ENST00000570924.1 | 0.81216076 | | 3.331158144 | 8.65E-04 |
| TGFB1 | ENST00000499732.1 | 0.8128901 | | 3.331008305 | 8.65E-04 |
| TGFB1 | ENST00000567913.2 | 0.80505034 | | 3.325440202 | 8.83E-04 |
| TGFB1 | NR_033338 | 0.80863007 | | 3.325333244 | 8.83E-04 |
| TGFB1 | NR_049762 | 0.80269935 | | 3.295658443 | 9.82E-04 |
| TGFB1 | ENST00000605014.1 | 0.80112112 | | 3.29510218 | 9.84E-04 |
| TGFB1 | NR_049761 | 0.80015514 | | 3.291252067 | 9.97E-04 |
| TGFB1 | ENST00000597755.1 | 0.80348345 | 3.291078391 | | 9.98E-04 |

**Supplementary Table 2.** **Clinical characteristics of the subjects for sample validation**

|  | **Smokers without COPD** | **COPD** |
| --- | --- | --- |
| **N** | 10 | 13 |
| **Sex, M/F^*^** | 9/1 | 11/2 |
| **Age, year^*^** | 56.28±11.09 | 64.45±10.78 |
| **BMI, kg/m^2*^** | 23.45±2.17 | 23.95±3.67 |
| **FEV1, %pred^#^** | 98.99±15.51 | 83.67±25.04 |
| **FEV1/FVC%^#^** | 81.93±9.31 | 64.14±6.54 |

*P>0.05, #P<0.05. Data are presented as the means ± SDs.

**Supplementary Table3.** **The genes in the intersection of the DEGs in *HSALR1* RNA-seq.**

**The intersection of up-regulated genes in si-NC+NC vs si-NC+TGF-β group and down-regulated genes in si-NC+TGF-β vs si-*HSALR1*+TGF-β group**

| **Gene Symbol** | |  | |  |  |  |
| --- | --- | --- | --- | --- | --- | --- |
| KCNH1 | EDN1 | | EGR2 | AMIGO2 | EFR3B | LRRC15 |
| KRT34 | TPM1 | | RTKN2 | SLFNL1 | ATP10A | LIMS2 |
| SGCG | CAP2 | | NRG1 | CDC45 | MAMDC2 | ADAM19 |
| CTPS1 | PCK2 | | CDH15 | C8orf4 | PAQR4 | DKK1 |
| SCN9A | BRIP1 | | POLQ | EXO1 | KIF14 | MKI67 |
| SCRG1 | ADAMTS6 | | TNFRSF9 | CLSPN | FGF14 | SLC7A5 |
| SDF2L1 | HECW2 | | LMO7 | HELLS | ULBP2 | PTPRE |
| E2F8 | XYLT1 | | CDC6 | CCNE2 | HHAT |  |

**The intersection of down-regulated genes in si-NC+NC vs si-NC+TGF-β group and down-regulated genes in si-NC+TGF-β vs si-*HSALR1*+TGF-β group**

| **Gene Symbol** | |  | |  |  |  |
| --- | --- | --- | --- | --- | --- | --- |
| SLC39A8 | DUSP4 | | PTCHD4 | RAPGEFL1 | PIANP | EPHB6 |
| MEF2C | TBX2 | | CEBPD | ADAMTS9 | ACBD4 | RMDN2 |
| DES | GPR162 | | IL1R1 | INPP5D | SLC1A3 | IL1RL1 |
| TRAF3IP2 | DENND2D | | ANGPT1 | RNF112 | PF4V1 | MMP23B |
| WFDC3 | CD1D | | BTBD11 | FOXG1 | TLE2 | TNFAIP2 |
| TYMSOS | PARK2 | | EFNB3 | CRABP2 | SLC16A6 | TCEA3 |
| DUSP10 | AXIN2 | | PIK3R3 | ZNF467 | CHL1 | CDKN1C |
| IKZF2 | KCNG2 | | PC | ANGPTL2 | C1QTNF1 | SECTM1 |
| LAMA4 | DTNA | | SOX5 | ABLIM1 | SFMBT2 | AMPH |
| PLPPR3 | A2M | | SCNN1A | EFNA1 | CXCL6 | VWA5A |
| SEMA3B | NR4A2 | | ENOX1 | FAM13C | ARHGAP6 | HPD |
| SOBP | COL9A3 | | CA11 | SGPP2 | PTGDR2 | SULF2 |
| NPR3 | CACNB2 | | PCDH18 | NPR1 | IL6R | HOTS |
| CNR1 | MAFB | | SNAP25 | YPEL4 | FGFR4 | CXCL12 |
| HR | MAP7D2 | | CYP3A7 | ITGB8 | C3 | MXRA5 |
| CIDEC | ENPP5 | | ELANE | INHBB | KCND2 | CYP3A7 |
| PPL | SRGAP3 | | SEMA6B | SIX2 | RORB | CHRM2 |

**Supplementary Table4.** **Primers used in experiments**

| **Primer name** | **Forward primer (5’-3’)** | **Reverse primer (5’-3’)** | **Application** |
| --- | --- | --- | --- |
| *HSALR1*  GAPDH  HSP90AB1 | GAGCAGCAGTCATCAGGGAAC  CAGCCTCAAGATCATCAGCA  ATTGTGACCAGCACCTACGG | TGCGATACACCTGACCTTGC  ACAGTCTTCTGGGTGGCAGT  CATGGTGGAGTTGTCCCGAA | qRT-PCR  qRT-PCR  qRT-PCR |
| Smad3 | GCCTGCTGGGCTGGA | ATGGGACACCTGCAACCG | qRT-PCR |
| NFKB1  HELLS | GCCTCCACAAGGCAGCAAATA  AGCGGTTGTGAGGAGTTAGC | CACCACTGGTCAGAGACTCGGTAA  CATGCCTGGACACTCACCC | qRT-PCR  qRT-PCR |
| ENST00000601801 | GCAGCAGCCCTGGAAATGAT | TGGCGCCTTAACTCCACATC | qRT-PCR |
| ENST00000572151 | TTTTCAGCGGATAGCTGAGGC | ATGGGGACTGAGCTGTTTCTT | qRT-PCR |
| ENST00000567913 | GAAGAGAGACTGACTGGGCAAC | CACCCCTCTGGAATCCACTTAT | qRT-PCR |
| E2F8 | GGACAGTACCTGCTTGCCTT | GGAGGTCGCTTGACAGGAAA | qRT-PCR |
| CDC6 | GCTGCCCTTAGATGAAGCCA | GCTGCCCTTAGATGAAGCCA | qRT-PCR |
| CLSPN | TAAACCACGGCTAGGTGCTG | CCAGAAACGCTGCTTCAAGG | qRT-PCR |
| CDC45 | GTGGCTATGTTCGTGTCCGA | AAGAGAAGGACCCTCTGGCT | qRT-PCR |
| PIK3R3 | CGGTCGGGTTGGTTCTTACA | CTGGTCTGCAGAGAGCGAAT | qRT-PCR |
| IL1R1 | GAGCGGCAGGAATGTGACAA | CAAGGGGTCCAGCTTCTCAG | qRT-PCR |
| IL6R | GGTCAAGGACCTCCAGCATC | AGAATCTTGCACTGGGAGGC | qRT-PCR |
| FGFR4 | TGAGGAGGAGCCAGGAAGG | GCTCAAGCTCCACTTCCTCA | qRT-PCR |
| LAMA4 | ACAGGCTGTGATAAGTGCGT | CCGGATTTGCCTTCCTCGAT | qRT-PCR |
| ITGB8 | TGAGGCGAAAAGGACAAGGG | TTAGTTGCAGCTTTGCAGCC | qRT-PCR |
| ANGPT1 | CCACAACCTTGTCAATCTTTGC | TTACAGTCCAACCTCCCCCA | qRT-PCR |
| Akt1  NFKB1  NEAT1  RELA  MAPK | GGAGGTTTTTGGGCTTGCG CATTGCCCATCGGGATGTC  GCCTCCACAAGGCAGCAAATA  CCAGTTTTCCGAGAACCAAA  CTTCCAAGAAGAGCAGCGTG | GTCCATGGTGTTCCTACCCA ACGCTTCACGAATTTGCGTGTC  TGGCAAAGCCAAAGTCAGTGAG  ATGCTGATCTGCTGCGTATG  GCCTGGTCCCGTGAAATACA | qRT-PCR  qRT-PCR  qRT-PCR  qRT-PCR  qRT-PCR |
| U1 | TCAAGAAGGATGCACCCCCA | ATAATACGCCCGAGTTCCCC | qRT-PCR |
| TGF-β1 | CTGTCCAACATGATCGTGCG | GACACAGAGATCCGCAGTCC | qRT-PCR |
| *HSALR1*pro-P1 | AACAAAGTTACCCTCGATGTTGG | CCCTTCTTCTGCCTCCTTCC | ChIP-qPCR |
| *HSALR1*pro-P2 | GCCACAGAGGGAACCTCACG | CCATGCAGTCGCCATCCTG | ChIP-qPCR |
| *HSALR1*pro-P3 | ACGCAGTGCTCCTTGTGTG | CATCTGGGCTAGGTAACGGC | ChIP-qPCR |
| sh-*HSALR1*-1 | GTGCTCCTTGTGTGACTCACA | TGTGAGTCACACAAGGAGCAC | shRNA |
| sh-*HSALR1*-2 | CTGCCTGACACCTTCCTACTT | AAGTAGGAAGGTGTCAGGCAG | shRNA |
| siHSP90AB1-1 | CAGTGAGCTTCGCTGATGA | TCATCAGCGAAGCTCACTG | siRNA |
| siHSP90AB1-2 | ACCACAAGATGGTGGACAA | TTGTCCACCATCTTGTGGT | siRNA |
| siHSP90AB1-3 | CCAGCCTCATCATCCTCAT | ATGAGGATGATGAGGCTGG | siRNA |
| siSmad3-1 | GGTGCTCCATCTCCTACTA | TAGTAGGAGATGGAGCACC | siRNA |
| siSmad3-3 | GCAACCTGAAGATCTTCAA | TTGAAGATCTTCAGGTTGC | siRNA |
